# Whole-transcriptome analysis of differentially expressed genes between ray and disc florets and identification of flowering regulatory genes in *Chrysanthemum morifolium*

**DOI:** 10.3389/fpls.2022.947331

**Published:** 2022-08-04

**Authors:** Hua Liu, Yin Jia, Yuhong Chai, Sen Wang, Haixia Chen, Xiumei Zhou, Conglin Huang, Shuang Guo, Dongliang Chen

**Affiliations:** ^1^Institute of Grassland, Flowers and Ecology, Beijing Academy of Agriculture and Forestry Sciences, Beijing, China; ^2^College of Landscape Architecture, Sichuan Agricultural University, Chengdu, China; ^3^School of Horticulture and Landscape Architecture, Henan Institute of Science and Technology, Xinxiang, China; ^4^Chengdu Park City Construction Development Research Institute, Chengdu, China

**Keywords:** *Chrysanthemum morifolium*, ray florets, disc florets, transcriptome, RNA-seq, differentially expressed genes

## Abstract

*Chrysanthemum morifolium* has ornamental and economic values. However, there has been minimal research on the morphology of the chrysanthemum florets and related genes. In this study, we used the leaves as a control to screen for differentially expressed genes between ray and disc florets in chrysanthemum flowers. A total of 8,359 genes were differentially expressed between the ray and disc florets, of which 3,005 were upregulated and 5,354 were downregulated in the disc florets. Important regulatory genes that control flower development and flowering determination were identified. Among them, we identified a *TM6* gene (*CmTM6-mu*) that belongs to the Class B floral homeotic MADS-box transcription factor family, which was specifically expressed in disc florets. We isolated this gene and found it was highly similar to other typical *TM6* lineage genes, but a single-base deletion at the 3′ end of the open reading frame caused a frame shift that generated a protein in which the TM6-specific paleoAP3 motif was missing at the C terminus. The *CmTM6-mu* gene was ectopically expressed in *Arabidopsis thaliana*. Petal and stamen developmental processes were unaffected in transgenic *A. thaliana* lines; however, the flowering time was earlier than in the wild-type control. Thus, the C-terminal of paleoAP3 appears to be necessary for the functional performance in regulating the development of petals or stamens and *CmTM6-mu* may be involved in the regulation of flowering time in chrysanthemum. The results of this study will be useful for future research on flowering molecular mechanisms and for the breeding of novel flower types.

## Introduction

As one of the most important cut flower species, *Chrysanthemum morifolium* is cultivated worldwide (Teixeira, 2003; Silva et al., 2013). Chrysanthemum flowers have capitula consisting of two types of florets (ray and disc), which are derived from one torus. Capitula are complex structures that include (from the outside to the inside) bracts, a torus, ray florets, and disc florets (Liu et al., 2020). The differentiation and development of ray and disc florets mediate the formation of various flower types in cultivated chrysanthemum varieties (Song et al., 2018). In the same genetic background, the two types of florets vary substantially in their position, fertility, symmetry, organ fusion, pigment composition (Berger et al., 2016; Novaković et al., 2019; Su et al., 2019). However, because of the lack of genome information, the molecular regulation mechanisms involved in the development of the two types of florets in chrysanthemum are still not fully understood.

In a previous study, Liu et al. identified homologs of important regulators of flower development and organ determination, including homologs of Class A, B, C, and E genes as well as two *MCMl/AGAMOUS/DEFICIENS/SERUM RESPONSE FACTOR* (*MADS*)-box genes (*WUSCL* and *KNUCL*) that were differentially expressed between the ray and disc florets, verifying their important roles in flower development and organ determination (Liu et al., 2016a). Indeed, many previous studies have proved that the expression of homologous genes related to floral organ development in higher plants vary in the flower heads of Asteraceae plants (Ren et al., 2013; Liu et al., 2015, 2016a). Among these genes, the *CYCLOIDEA2* (*CYC2*)-like genes, as well as the Class B and Class C *MADS*-box genes, may be involved in regulating the distinct development of the two types of florets in *Asteraceae* (Burke, 2008; Chapman et al., 2012). Currently, among higher plants, floral developmental and regulatory activities have been characterized primarily in *A. thaliana*, *Antirrhinum majus*, and other model species (Johannesson et al., 2001; Clark and Coen, 2002; Juntheikki-Palovaara et al., 2014).

Class B genes also help determine petal and stamen development (Mahajan and Yadav, 2014; Sasaki et al., 2014; Prunet et al., 2017). There are two Class B gene lineages, *PISTILLATA* (*PI*)/*GLOBOSA* (*GLO*) and *APETALA3* (*AP3*)/*DEFICIENS* (*DEF*)/*Tomato MADS-BOX GENE 6* (*TM6*; Kramer et al., 1998; Vandenbussche et al., 2003; Zhang et al., 2011). The *AP3*/*DEF*/*TM6* lineage is further divided into two sublineages (i.e., *AP3* and *TM6*; Vandenbussche et al., 2003). The *AP3* and *TM6* genes encode proteins that have euAP3 and paleoAP3 motifs, respectively, in the C-terminal regions (Kramer et al., 2006; Ackerman et al., 2008). The *AP3* lineage genes mediate petal and stamen development, whereas the *TM6* lineage genes mostly influence stamen development (Tsaftaris et al., 2006; Yeoh et al., 2016; Martín-Pizarro et al., 2018). However, the genetic regulatory mechanism underlying the development of chrysanthemum capitula remains unknown. Elucidating the mechanism controlling the development of the two types of chrysanthemum florets in the same genetic background is critical for the breeding of chrysanthemum varieties with enhanced flower types.

Flowering time is an important ornamental trait for flowering plants. Six major floral induction pathways have been identified in *A. thaliana*, and these pathways converge to regulate a small number of “floral integrator genes,” such as, *FLOWERING LOCUS T* (*FT*) and *SUPPRESSOR OF OVEREXPRESSION OF CONSTANS 1* (*SOC1*), both of which rapidly promote floral development (Fornara et al., 2010). Chrysanthemum is a typical short-day flowering plant and floral induction is controlled mainly through the photoperiod pathway (Oda et al., 2017). As was found in model plants, in chrysanthemum, *CONSTANS* (*CO*) is the core gene of the photoperiod pathway, which is induced by circadian clock genes, such as *CRYPTOCHROMES* (*CRY*s) and *GIGANTEA* (*GI*), and activates *FT* transcription in leaves (Yang et al., 2017; Sun et al., 2018; Oda et al., 2020). The balance between *FT* and an ortholog of *FT*, *ANTI-FLORIGENIC FT/TFL1 FAMILY PROTEIN* (*AFT*) determines the progression of floral transition and anthesis (Higuchi et al., 2013; Oda et al., 2020).

Other floral induction pathways, such as the gibberellin (GA), age, and ambient temperature pathways, have been found in chrysanthemum (Sumitomo et al., 2009; Gao et al., 2017; Wei et al., 2017). The ambient temperature pathway converges to regulate the expression of *FT* by *SHORT VEGETATIVE PHASE* (*SVP*). Overexpression of the *C. morifolium SVP* gene delayed blossoming in transgenic *Arabidopsis*, indicating that a similar ambient temperature pathway may exist in chrysanthemum (Gao et al., 2017). The GA and age pathways were shown to converge to regulate the expression of *SOC1*. In chrysanthemum, the nuclear factor Y, subunit B8 gene (*CmNFYB8*) was shown to influence flowering time by directly regulating the expression of the microRNA *cmo-MIR156* in the aging pathway (Wei et al., 2017). GA was found to promote flowering in chrysanthemum by up-regulating *FLORICAULA/LEAFY* (*FL*) expression (Sumitomo et al., 2009). Flowering regulation is a complex biochemical process that involves a large number of genes and complex molecular regulation networks. Research on the flowering regulation mechanism has important implications for the accurate regulation of flowering time.

Transcriptome sequencing is important for investigating floral organ development in higher plants because it enables the rapid and efficient screening of many differentially expressed genes (DEGs; Liu et al., 2017; Pei et al., 2017). In floral development research, transcriptome sequencing technology has been widely used for basic screening work (Fu et al., 2018). For example, an earlier transcriptome analysis identified numerous MADS-box genes that are differentially expressed between wild-type (WT) and lip-flap *Phalaenopsis* mutants, revealing how the orchid labellum differs and why the petal or sepal converts to a labellum in *Phalaenopsis* (Huang et al., 2015). Another transcriptome analysis, which examined the flowering transformation process in sugar apple, revealed the differential expression of genes related to circadian rhythms and plant signal transduction (Liu et al., 2016b). The transcriptome sequencing of the flower–fruit transformation process of grape trees and other fruit trees identified DEGs related to sugar and hormone signaling pathways (Domingos et al., 2016). Transcriptome sequencing techniques have been used to investigate various stages of floral development in plants such as broccoli, orchid, and grape (Huang et al., 2015; Domingos et al., 2016; Liu et al., 2016a, 2017; Pei et al., 2017; Fu et al., 2018). Therefore, in this study, we applied high-throughput sequencing technology to analyze genes that are differentially expressed between chrysanthemum ray and disc florets.

The transcriptome sequencing and comparative analysis of chrysanthemum ray florets, disc florets, and leaves were performed using Illumina sequencing technology and RNA sequencing (RNA-seq) data. We identified DEGs between ray florets and disc florets to reveal important regulators controlling the differential development of the two floret types. Next, we identified important regulatory genes that may be involved in controlling floral development and floral organ identity. A list was compiled of candidate genes for future functional analyses of flowering regulation in chrysanthemum. We also isolated a *TM6* homolog in *C. morifolium* (*CmTM6-mu*). This gene shares a high similarity with other *TM6* lineage genes (Ackerman et al., 2008; Yeoh et al., 2016; Martín-Pizarro et al., 2018), but a single-base deletion was observed at the 3′ terminal of the open reading frame (ORF), which caused a frameshift and led to the encoding product not having the typical paleoAP3 motif at its C-terminal. The gene function of *CmTM6-mu* was analyzed by ectopic expression in *A. thaliana*. Transgenic *Arabidopsis* showed no petals or stamen developmental affects, as expected; however, an earlier flowering phenotype was observed in almost all the transgenic lines, which indicated that the C-terminus of paleoAP3 is necessary for the functional performance in regulating the development of petals or stamens and that *CmTM6-mu* may be involved in the regulation of flowering time in chrysanthemum.

Our results will provide researchers with valuable genomic information and candidate genes that are potentially useful for flowering molecular mechanism studies and for the breeding of novel flower types. Furthermore, the data presented herein may be useful for clarifying the molecular mechanisms regulating the differential development of ray and disc florets in chrysanthemum flowers.

## Results

### Screening, comparison, and analysis of data

**Figure 1:**
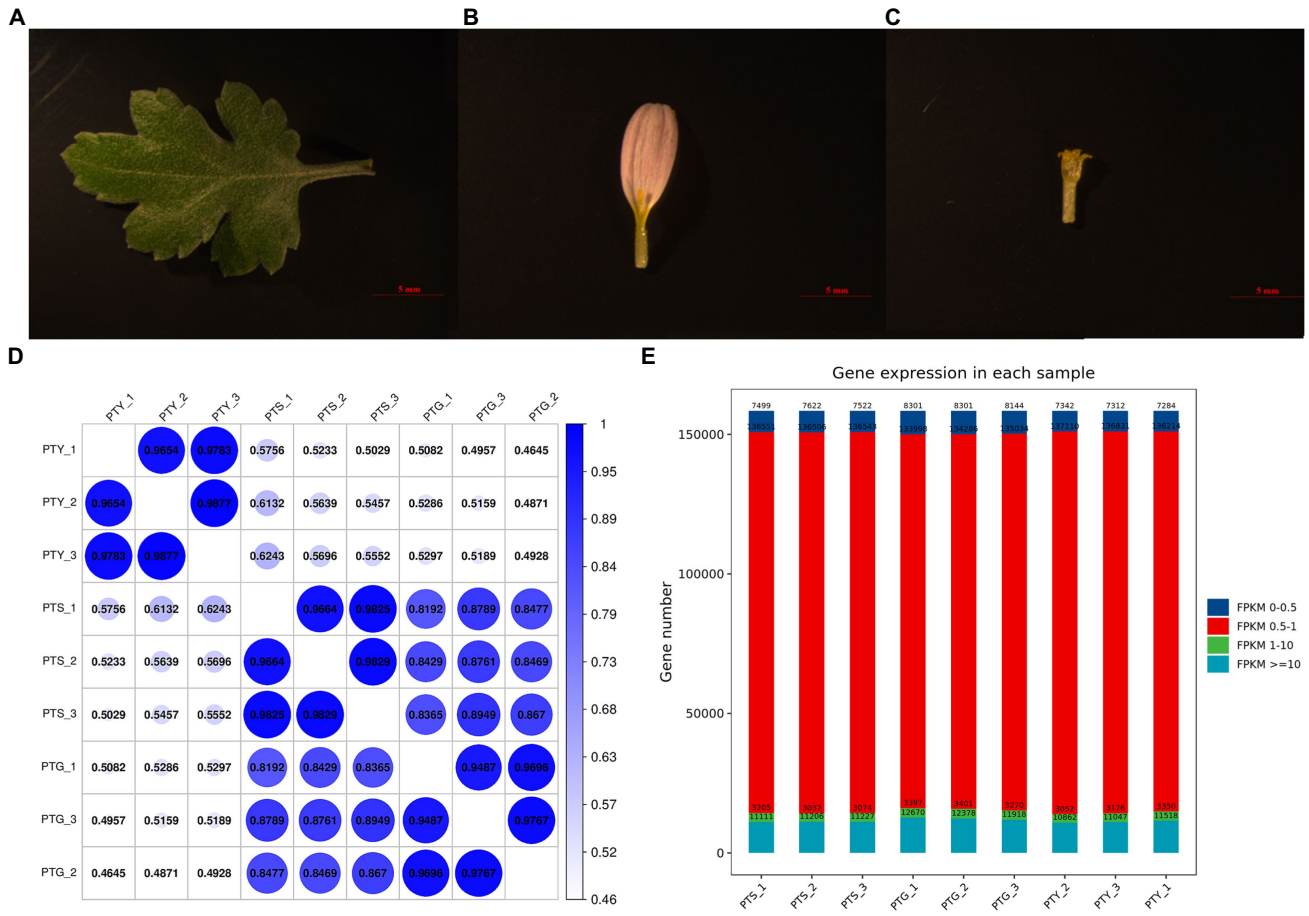
Chrysanthemum samples used for transcriptome sequencing and gene expression levels and correlation analysis results. **(A–C)** Leaf, ray floret, and disc floret, respectively, of *Chrysanthemum morifolium* Ramat “Pink Carpet.” **(D)** Gene expression levels in each sample. **(E)** Gene expression level statistics of each sample.

Total RNA was extracted from the ray and disc florets of the capitula, as well as the fully extended leaves of chrysanthemum cultivar “Pink Carpet” during the full-bloom stage (Figures 1A–C). The RNA was used to construct nine libraries (i.e., three biological replicates per sample type) for the RNA-seq analysis. Approximately 38–58 million reads were generated per library. After a strict quality control step during which low-quality data were eliminated, ~37–56 million clean reads were retained for each library. Details regarding the sequencing data are provided in Table 1. For each sample, more than 96% of the raw reads were retained as clean reads for all three replicates. Thus, the high-quality sequencing data were appropriate for the subsequent analyses. The HISAT2 program was used to align the clean reads to the *Chrysanthemum seticuspe* reference genome (Kim et al., 2015). The mapping rates for all samples exceeded 75% (Table 1). Therefore, the sequenced plants were closely related to *C. seticuspe*.

**Table 1:** General statistics for the Illumina HiSeq 2000 sequencing data.

Sample	Raw reads	Clean reads	Clean reads rate (%)	Q30 (%)	GC%	Total mapped reads	Mapping rates (%)
PTS-1	57.75M	56,327,280	96.60	91.87	43.11	43,007,305	76.35
PTS-2	50.60M	49,240,032	96.31	91.97	42.68	37,374,818	75.90
PTS-3	55.91M	54,365,902	96.20	91.75	42.94	41,319,419	76.00
PTG-1	53.48M	51,929,312	96.18	91.73	42.86	40,045,922	77.12
PTG-2	57.80M	56,192,456	96.29	91.85	42.68	42,497,308	75.63
PTG-3	58.05M	56,858,672	97.12	92.26	43.33	44,422,517	78.13
PTY-1	38.24M	37,373,262	96.51	92.44	42.63	28,976,082	77.53
PTY-2	50.28M	48,827,632	96.17	91.53	43.10	38,096,697	78.02
PTY-3	56.43M	55,116,590	96.75	92.67	43.24	42,984,714	77.99

PTS, ray florets; PTG, disc florets; PTY, leaves.

Using known reference gene sequences and annotation files, sequence similarities were determined, and the expression levels of each protein-coding gene in each sample were determined. The fragments per kilobase of transcript per million mapped reads (FPKM) values calculated using the Cufflinks software were used to represent gene expression levels. The gene expression levels were highly correlated among the three biological replicates for the different sample types (Figure 1D), implying that the sequencing data were reliable. The average FPKM value for the three biological replicates was calculated for each gene in each sample. Most gene expression levels were between 0.5 and 1 (Figure 1E).

### Gene annotation and functional classification

A total of 150,346 unigenes were annotated following a BLAST search of seven databases [non-redundant (NR) protein database, Swiss-Prot, Gene Ontology (GO), Kyoto Encyclopedia of Genes and Genomes (KEGG), EuKaryotic Orthologous Groups (KOG), evolutionary genealogy of genes: Non-supervised Orthologous Groups (eggNOG), and Pfam], leaving 8,021 (5.06%) unannotated unigenes. More specifically, 150,122, 62,321, 55,481, 78,597, 18,304, 93,361, and 87,286 unigenes were annotated on the basis of information in the NR, Swiss-Prot, GO, KOG, KEGG, eggNOG, and Pfam databases, respectively.

**Figure 2:**
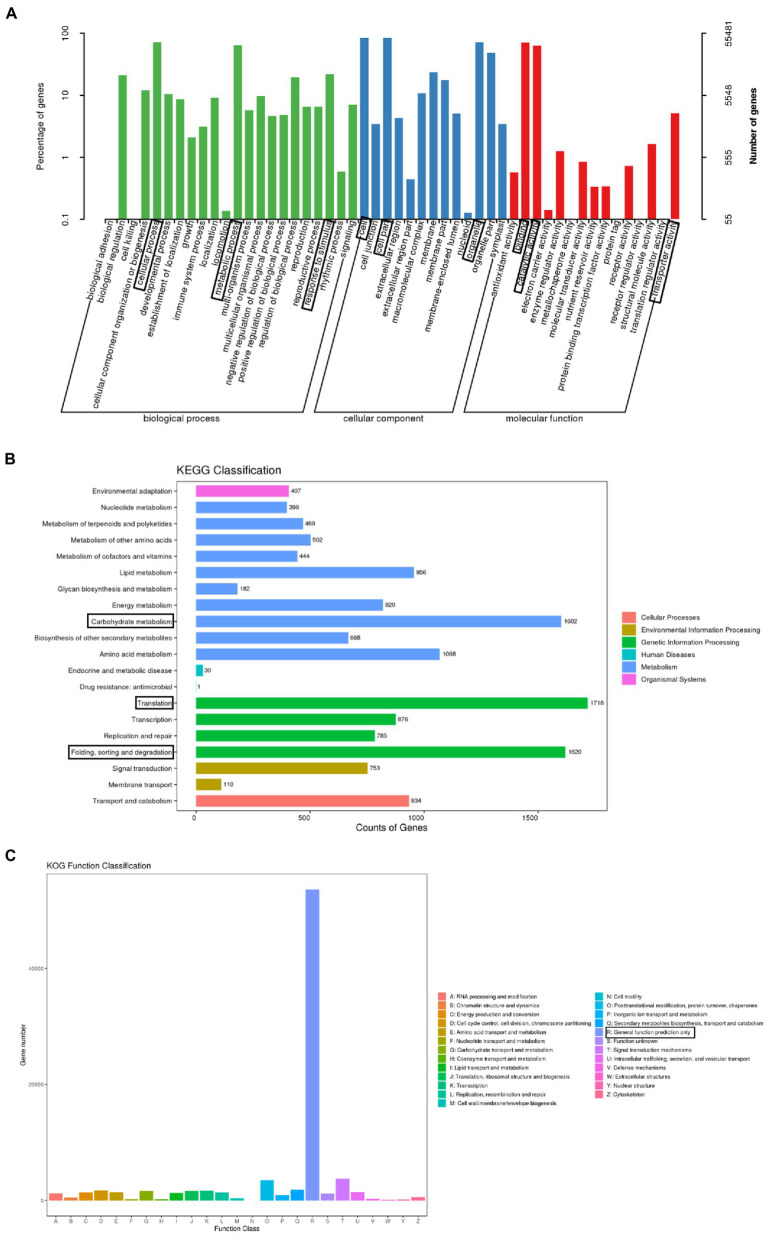
Functional annotations assigned to the unigenes. **(A)** Enriched gene ontology (GO) terms under the three main categories: biological process, molecular function, and cellular component. The three most significant GO terms in each category are boxed. **(B)** Enriched Kyoto Encyclopedia of Genes and Genomes (KEGG) pathways (level 2) in the six KEGG categories. The three most significant pathways are boxed. **(C)** Enriched EuKaryotic Orthologous Groups (KOG) functional classifications of identified paralogous proteins. The most significant classification is boxed.

The GO database is divided into the following three main categories: biological process, molecular function, and cellular component. A total of 55,481 unigenes were classified into 50 categories: 22 biological process, 13 cellular component, and 15 molecular function categories. The predominant biological process GO terms among the unigenes were “cellular process” and “metabolic process,” whereas “biological adhesion,” “cell killing,” and “locomotion” were relatively uncommon GO terms. The main cellular component GO terms among the unigenes were “cell,” “organelle,” and “cell part,” whereas “nucleoid” was a relatively uncommon GO term. The predominant molecular function GO terms among the unigenes were “binding” and “catalytic activity,” whereas “transcription regulator activity,” “protein tag,” “metallochaperone activity,” “electron carrier activity,” and “receptor regulator activity” were relatively uncommon GO terms (Figure 2A).

Under the biological process category, a total of 935 unigenes were annotated with at least one of the 8 terms were related to flowering time. They includes “photoperiodism, flowering” (499 genes), “long-day photoperiodism, flowering” (272 genes), “regulation of long-day photoperiodism, flowering” (72 genes), “regulation of photoperiodism, flowering” (42 genes), “short-day photoperiodism, flowering” (22 genes), “negative regulation of long-day photoperiodism, flowering” (17 genes), “negative regulation of short-day photoperiodism, flowering” (eight genes), and “positive regulation of short-day photoperiodism, flowering” (three genes; Table 2).

**Table 2:** Gene ontology (GO) biological process terms related to flowering time assigned to the unigenes.

GO ID	GO term	GO category	Number of the genes
GO:0048366 GO:0009585 GO:0009718 GO:0042753 GO:0010099 GO:0043153	Photoperiodism, flowering	Biological process	499
GO:0006325 GO:0048235 GO:0043433	Long-day photoperiodism, flowering	Biological process	272
GO:0048586 GO:0018107 GO:0010476	Regulation of long-day photoperiodism, flowering	Biological process	72
GO:1902326 GO:0080186 GO:0009648 GO:0090227 GO:0090239 GO:0010151 GO:0080050 GO:0043610	Regulation of photoperiodism, flowering	Biological process	42
GO:0048575 GO:0048572	Short-day photoperiodism, flowering	Biological process	22
GO:0009299	Negative regulation of long-day photoperiodism, flowering	Biological process	17
GO:0048577	Negative regulation of short-day photoperiodism, flowering	Biological process	8
GO:0048576	Positive regulation of short-day photoperiodism, flowering	Biological process	3

The KEGG database divides biological pathways into six categories, each of which is subdivided and displayed in a pathway graph that presents the molecular interaction networks in cells and in particular organisms. Of the 150,346 unigenes, 18,304 were assigned to at least one KEGG pathway, for a total of 127 KEGG pathways. The most represented pathways were “translation” (1,718 unigenes), “folding, sorting and degradation” (1,620 unigenes), and “carbohydrate metabolism” (1,602 unigenes), followed by “amino acid metabolism” (1,068 unigenes), “lipid metabolism” (956 unigenes), and “transport and catabolism” (934 unigenes). Additionally, 876 unigenes were associated with the “transcription” pathway (Figure 2B).

The KOG database is used to identify paralogous proteins. Gene annotation information was used to screen the KOG database. Among the 25 KOG categories, “general function prediction only” (53,642 unigenes) was the largest, followed by “signal transduction mechanisms” (3,087 unigenes) and “posttranslational modification, protein turnover, chaperones” (2,987 unigenes). In contrast, “cell motility” (five unigenes), “extracellular structures” (five unigenes), and “nuclear structure” (seven unigenes) were the smallest categories (Figure 2C).

### Differential gene expression analysis

**Figure 3:**
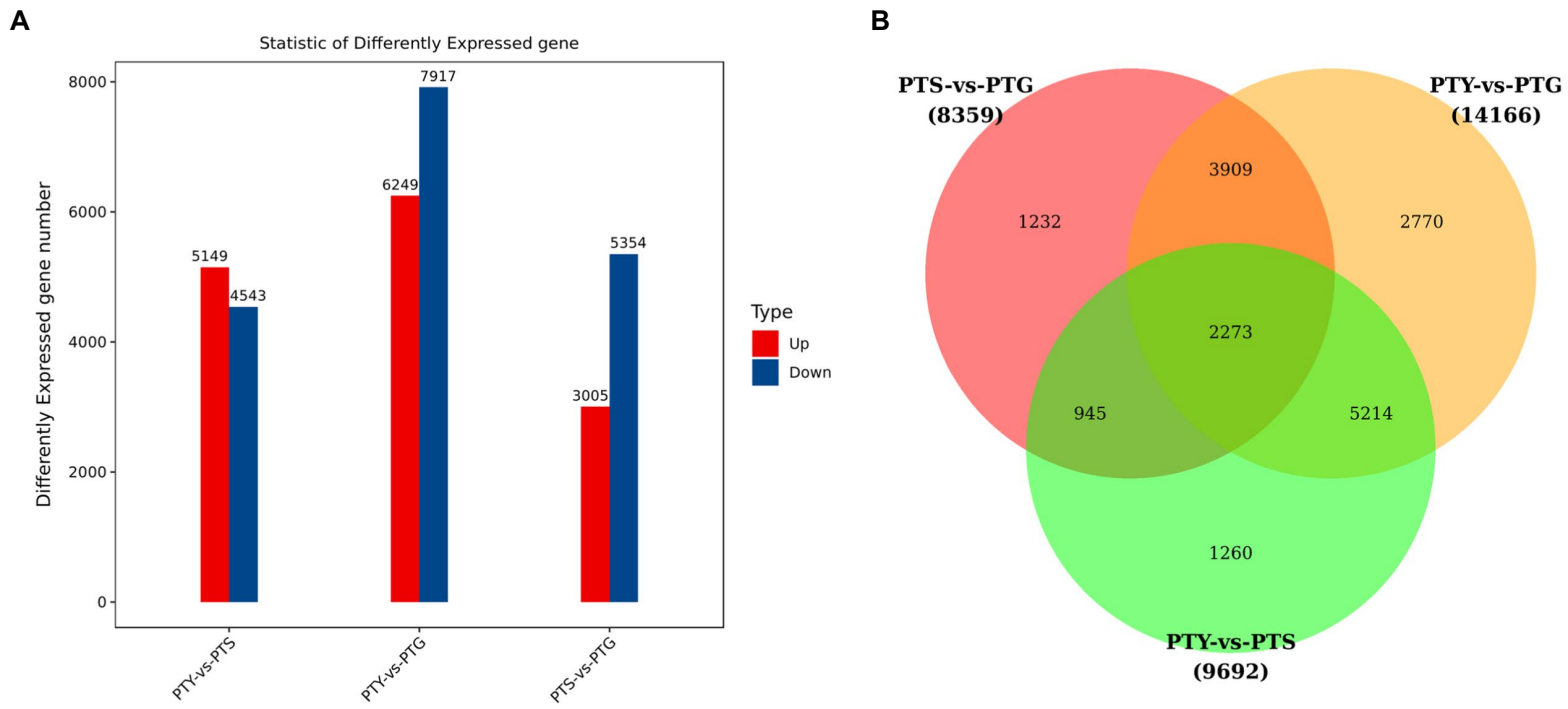
Differential gene expression analysis of leaves, disc florets, and ray florets in chrysanthemum. **(A)** Differentially expressed genes among the analyzed chrysanthemum samples. **(B)** Common and unique differentially expressed genes among the analyzed chrysanthemum samples.

Analyzing the differential expressions of genes between samples provides the basis for subsequent analyses of gene functions. We screened the DEGs among the ray florets, disc florets, and leaves using the following criteria: fold-change >2 and value of *p* <0.05. Accordingly, 8,359 genes were differentially expressed between ray florets and disc florets, of which the expression levels of 3,005 and 5,354 genes were higher and lower in the disc florets than in the ray florets, respectively (Figure 3A; Supplementary File 1). Moreover, 1,232 genes were differentially expressed specifically between the ray florets and disc florets (Figure 3B).

### Enriched gene ontology terms among the differentially expressed genes

The DEGs were screened for significantly enriched GO functions. Following the annotation of the DEGs with GO terms, the GO classifications were visualized using the WEGO software. The ray florets, disc florets, and leaves were analyzed to reveal the significantly enriched GO terms among the DEGs.

**Figure 4:**
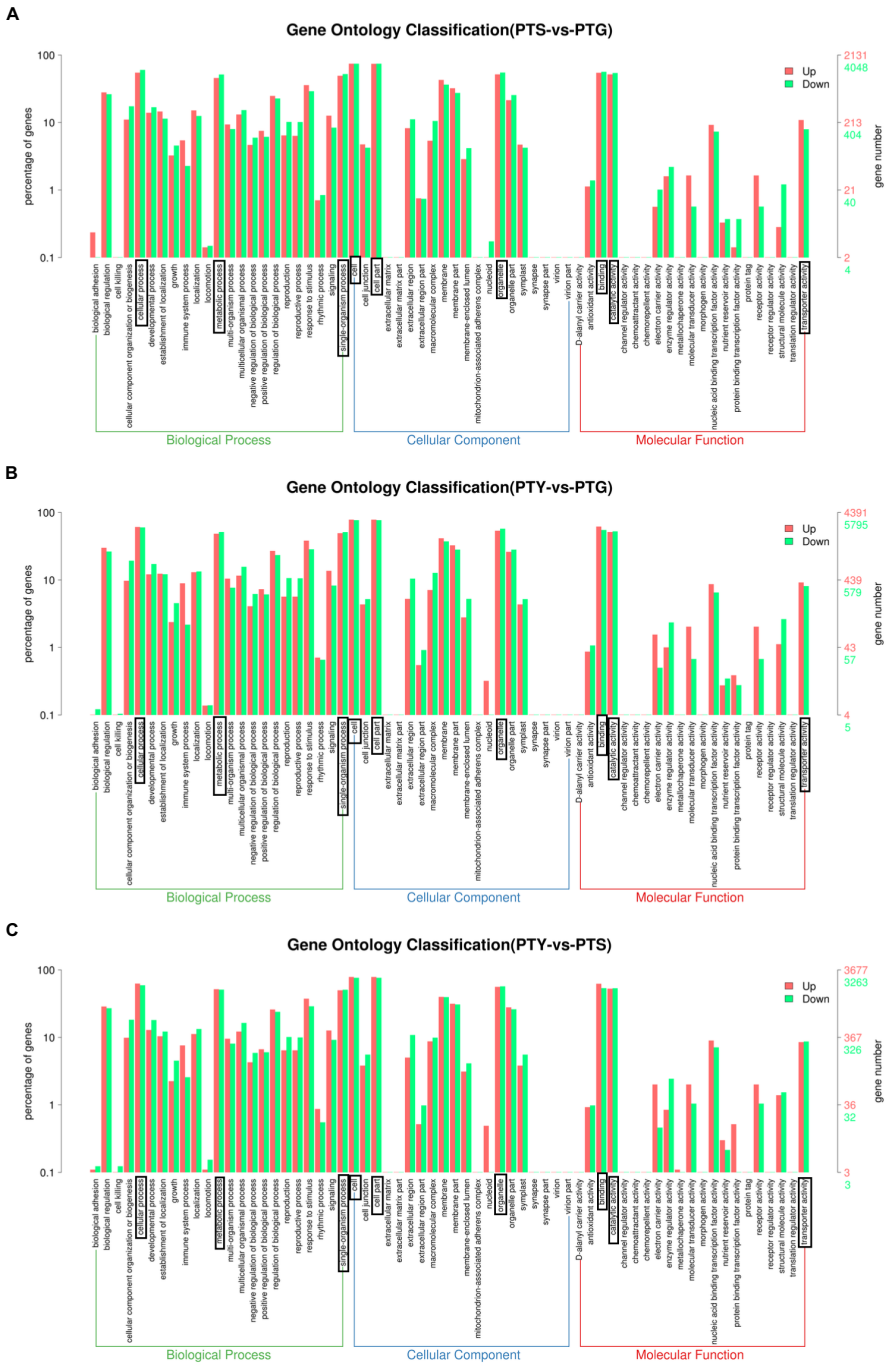
Enriched gene ontology (GO) terms assigned to the differentially expressed genes in comparisons among the leaf, disc floret, and ray floret samples. **(A–C)** Comparisons between ray florets and disc florets, leaves and disc florets, and leaves and ray florets, respectively. The three most significant GO classifications in each comparison are boxed. PTS, ray florets; PTG, disc florets; PTY, leaves.

A total of 3,989 GO terms were enriched among the DEGs between the ray florets and disc florets (Supplementary File 2). The most enriched GO terms were mainly related to cellular components (GO:0016021, GO:0005886, and GO:0005634) and molecular functions (GO:0046872 and GO:0005524). In the biological process category, many DEGs were related to “cellular processes,” “metabolic processes,” and “single biological processes.” In the cellular component category, the DEGs were mainly related to “cells,” “cell parts,” “organelles,” and “cell membranes.” In the molecular function category, most of the DEGs were related to “binding function” and “catalytic activity” (Figure 4A).

An analysis of the significantly enriched GO terms among the DEGs between the disc florets and leaves (Figure 4B) indicated that the distribution of significantly enriched GO terms was similar in the disc florets vs. leaves and disc florets vs. ray florets comparisons. The significantly enriched GO terms and their distribution were also determined for the DEGs between the ray florets and leaves (Figure 4C). The significantly enriched GO terms and the distribution of GO functions were essentially the same for the ray and disc florets.

### Significantly enriched pathways among differentially expressed genes

**Figure 5:**
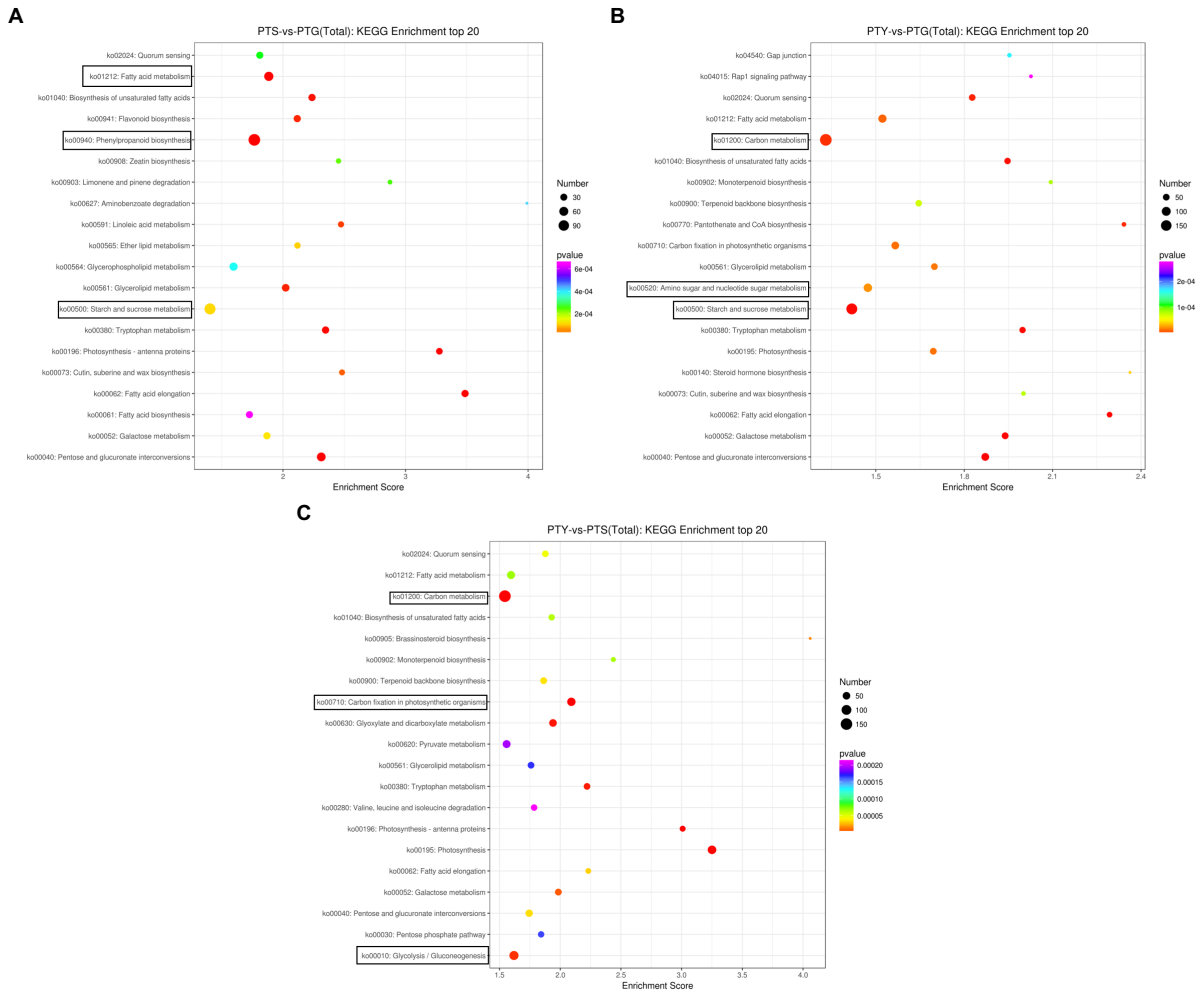
Bubble diagrams of enriched Kyoto Encyclopedia of Genes and Genomes (KEGG) pathways assigned to the differentially expressed genes in comparisons among the leaf, disc floret, and ray floret samples. **(A–C)** Comparisons between ray florets and disc florets, leaves and disc florets, and leaves and ray florets, respectively. The three most significant KEGG pathways in each comparison are boxed. PTS, ray florets; PTG, disc florets; PTY, leaves.

Biological functions *in vivo* require the coordinated expression of various genes. Pathway analyses are useful for clarifying the biological functions of genes. We used the KEGG database to determine the enriched pathways assigned to the protein-coding genes differentially expressed among the ray florets, disc florets, and leaves (Figure 5). A total of 190 KEGG pathways were enriched among the DEGs between the ray florets and disc florets, including “synthesis” (ko00940), “starch and sucrose metabolism” (ko00500), “plant hormone signal transduction” (ko04075), and “carbon metabolism” (ko01200; Supplementary File 3). The DEGs were mainly related to 20 metabolic pathways (Figure 5A). The most enriched pathway was “phenylpropanoid biosynthesis,” followed by “starch and sucrose metabolism,” “fatty acid metabolism,” and “pentose and glucose.” These genes may be associated with the conversion of uronic acid esters.

Of the top 20 enriched KEGG pathways among the DEGs between the disc florets and leaves, “carbon metabolism” and “starch and sucrose metabolism” were the main pathways (Figure 5B). In contrast, “carbon metabolism,” “glycolysis,” “photosynthesis,” and “fatty acid metabolism” were the primary enriched KEGG pathways among the DEGs between the ray florets and leaves (Figure 5C).

### Analysis of important differentially expressed genes

Bioinformatics analyses of the significantly enriched GO terms and KEGG pathways may help clarify the functions and metabolic pathways associated with the DEGs. However, to identify the key regulatory genes among the DEGs, we conducted a detailed analysis of the DEGs related to floral development. The results suggested that most of the DEGs are involved in inducing the flowering pathway, activating transcription factors (TFs), and regulating floral organ development and floral symmetry. This study mainly focused on floral organ development and the homologous genes related to the regulation of floral development and floral symmetry. The results of the analysis are listed in Table 3.

**Table 3:** Floral development-related differentially expressed genes in comparisons among the leaf, disc floret, and ray floret samples.

Homologous gene	Gene ID	Function notes	GO_number	FPKM-PTS	FPKM-PTG	FPKM-PTY
*CYC*	Cse_sc015869.1	Transcription factor CYCLOIDEA	GO:0003677, GO:0005634, GO:0007275	3.42	1.90	0
	Cse_sc027234.1	Transcription factor CYCLOIDEA	GO:0003677, GO:0005634, GO:0007275	2.45	2.11	0.21
	Cse_sc005798.1	Transcription factor CYCLOIDEA	GO:0003677, GO:0003700, GO:0005634, GO:0009799, GO:0009908, GO:0048262	5.66	0.62	0
*AP2*	Cse_sc005454.1	Floral homeotic protein APETALA 2	GO:0003677, GO:0003700, GO:0005634, GO:0009908, GO:0010073, GO:0010093, GO:0030154, GO:0048316, GO:0048481	11.64	22.07	4.56
	Cse_sc005866.1	Plant ovule development	GO:0003677, GO:0003700, GO:0005634, GO:0009908, GO:0010073, GO:0010093, GO:0030154, GO:0048316, GO:0048481	14.48	9.02	7.44
	Cse_sc010431.1	Meristem maintenance; flower development	GO:0003677, GO:0003700, GO:0005634, GO:0009908, GO:0010073, GO:0010093, GO:0030154, GO:0048316, GO:0048481	1.35	2.07	1.19
	Cse_sc010656.1	Specification of floral organ identity	GO:0003677, GO:0003700, GO:0005634, GO:0009908, GO:0010073, GO:0010093, GO:0030154, GO:0048316, GO:0048481	2.18	1.30	1.21
*AG2*	Cse_sc000289.1	Floral homeotic protein AGAMOUS	GO:0000977, GO:0003700, GO:0005634, GO:0045944, GO:0046983	2.40	23.92	0.32
	Cse_sc033361.1	Floral homeotic protein AGAMOUS	GO:0000977, GO:0003700, GO:0005634, GO:0045944, GO:0046983	6.29	210.90	0.08
*SOC1*	Cse_sc014932.1	MADS-box protein SOC1; vernalization response	GO:0000060, GO:0000977, GO:0000982, GO:0003700, GO:0005634, GO:0005737, GO:0007275, GO:0008134, GO:0009409, GO:0009739, GO:0009908, GO:0009909, GO:0009911, GO:0010048, GO:0010077, GO:0030154, GO:0043565, GO:0044212, GO:0045893, GO:0045944, GO:0046983	0.40	0.49	17.23
	Cse_sc017768.1	MADS-box protein SOC1	GO:0000060, GO:0000977, GO:0000982, GO:0003700, GO:0005634, GO:0005737, GO:0007275, GO:0008134, GO:0009409, GO:0009739, GO:0009908, GO:0009909, GO:0009911, GO:0010048, GO:0010077, GO:0030154, GO:0043565, GO:0044212, GO:0045893, GO:0045944, GO:0046983	0.51	3.54	27.59
	Cse_sc029815.1	MADS-box protein SOC1	GO:0000060, GO:0000977, GO:0000982, GO:0003700, GO:0005634, GO:0005737, GO:0007275, GO:0008134, GO:0009409, GO:0009739, GO:0009908, GO:0009909, GO:0009911, GO:0010048, GO:0010077, GO:0030154, GO:0043565, GO:0044212, GO:0045893, GO:0045944, GO:0046983	0.57	1.06	12.82
*TCP2*	Cse_sc000095.1	Transcription factor TCP2	GO:0003700, GO:0005634, GO:0006355, GO:0009637, GO:0009965, GO:0030154, GO:0043565, GO:0045962, GO:0048366, GO:1903508, GO:2000306	7.09	3.28	10.36
	Cse_sc011954.1	Transcription factor TCP2	GO:0003700, GO:0005634, GO:0006355, GO:0009637, GO:0009965, GO:0030154, GO:0043565, GO:0045962, GO:0048366, GO:1903508, GO:2000306	19.49	14.25	26.90
*MYB1*	Cse_sc009852.1	Transcription factor MYB1; DNA binding	GO:0003677, GO:0003700, GO:0005634, GO:0009751	1.74	2.37	1.51
	Cse_sc040226.1	Transcription factor MYB1	GO:0003677, GO:0003700, GO:0005634, GO:0009751	0.08	0.26	0.10
	Cse_sc047413.1	Transcription factor MYB1	GO:0003677, GO:0003700, GO:0005634, GO:0009751	0.63	0	0
*DIV*	Cse_sc002812.1	Flower development	GO:0003677, GO:0005634, GO:0009908, GO:0048262	1.02	0.35	2.76
	Cse_sc017952.1	Determination of dorsal;ventral asymmetry	GO:0003677, GO:0005634, GO:0009908, GO:0048262	4.84	14.48	2.43
	Cse_sc033590.1	Flower development	GO:0003677, GO:0005634, GO:0009908, GO:0048262	12.09	14.33	9.92
	Cse_sc034623.1	Transcription factor DIVARICATA	GO:0003677, GO:0005634, GO:0009908, GO:0048262	2.73	5.61	4.23
	Cse_sc035745.1	Transcription factor DIVARICATA	GO:0003677, GO:0005634, GO:0009908, GO:0048262	0.14	0.60	0
*MADS*	Cse_sc001511.1	MADS-box protein; regulation of meristem development	GO:0000977, GO:0000982, GO:0003677, GO:0005634, GO:0007275, GO:0008134, GO:0010022, GO:0010093, GO:0010582, GO:0030154, GO:0043565, GO:0044212, GO:0045944, GO:0046983, GO:0048509	3.37	3.83	2.31
	Cse_sc013015.1	MADS-box protein	GO:0000977, GO:0000982, GO:0005634, GO:0007275, GO:0008134, GO:0009908, GO:0030154, GO:0043565, GO:0044212, GO:0045944, GO:0046983	1.82	1.71	0.70
	Cse_sc001511.1	MADS-box protein	GO:0000977, GO:0003700, GO:0005634, GO:0045944, GO:0046983, GO:0048481	15.52	10.95	11.34
	Cse_sc015618.1	MADS-box protein	GO:0000977, GO:0000982, GO:0003677, GO:0005634, GO:0007275, GO:0008134, GO:0009553, GO:0010022, GO:0010093, GO:0010094, GO:0010582, GO:0030154, GO:0043565, GO:0044212, GO:0045893, GO:0045944, GO:0046983, GO:0048316, GO:0048437, GO:0048449, GO:0048455, GO:0048457, GO:0048459, GO:0048481, GO:0048509, GO:0048833, GO:0080060, GO:0080112	43.44	32.12	0.61
	Cse_sc006236.1	MADS-box protein	GO:0000977, GO:0003700, GO:0005634, GO:0040008, GO:0045944, GO:0046983	0.06	0.13	0.47
	Cse_sc042534.1	Floral homeotic protein PMADS 1	GO:0000977, GO:0003700, GO:0005634, GO:0007275, GO:0045944, GO:0046983	96.90	70.63	0.15
	Cse_sc000306.1	MADS-box protein GDEF (TM6)	GO:0000977, GO:0003700, GO:0005634, GO:0007275, GO:0045944, GO:0046983	0.11	3.30	0
	Cse_sc005132.1	Floral homeotic protein PMADS 2	GO:0000977, GO:0003700, GO:0005634, GO:0007275, GO:0045944, GO:0046983	637.52	1318.94	10.78

PTS, ray florets; PTG, disc florets; PTY, leaves.

On the basis of the annotation information and sequence analyses, the key genes related to the regulation of floral organ development, *APETALA2* (*AP2*) and *AGAMOUS2* (*AG2*), were identified in the transcriptome (Becker and Theissen, 2003; Aida et al., 2008; Wollmann et al., 2010). Moreover, *TEOSINTE BRANCHED1*/*CYCLOIDEA* /*PROLIFERATING CELL FACTOR* (*TCP*), *V-MYB AVIAN MYELOBLASTOSIS VIRAL ONCOGENE HOMOLOG* (*MYB*), and *MADS* genes, which help regulate flowering, meristem differentiation, and floral symmetry, were also identified, as were *CYC*, *DIVARICATA* (*DIV*), and other homologous genes (Galego and Almeida, 2002; Dornelas et al., 2011; Huang and Irish, 2015; Niwa et al., 2018; Victoria and Minsung, 2017).

TFs are important for plant development at the cellular, tissue, and organ levels (Shin et al., 2002; Hileman, 2014; Lai et al., 2020). Previous studies proved that the TCP, MYB, and MADS TF families, as well as TFs from other families, have key regulatory functions that influence floral development (Kaufmann et al., 2009; Hileman, 2014; Ke et al., 2021; Li et al., 2021). Our RNA-seq analysis revealed that the *TCP2*, *MYB1* (Huang and Irish, 2015; Niwa et al., 2018), and *MADS* gene expression levels varied significantly among the ray florets, disc florets, and leaves. Additionally, expression-level differences were detected for multiple *CYC* and *DIV* genes that regulate floral symmetry. Significant differences in gene expression were also observed between the two floret types. Most *TCP* family genes and *CYC* genes were significantly more highly expressed in the ray florets than in the disc florets, whereas the opposite pattern was detected for the *AG* and *SOC* family genes (Wollmann et al., 2010; Li et al., 2021). Some *AP*, *MYB*, *DIV*, and *MADS* family genes were more highly expressed in the ray florets than in the disc florets, but some were more highly expressed in disc florets. These findings imply that changes in the expression levels of floral development-related TFs may cause meristems to differentiate in different directions. These genes should be investigated more thoroughly regarding their effects on chrysanthemum floret development.

### Identification of important genes and signaling pathways involved in flowering control in chrysanthemum

**Figure 6:**
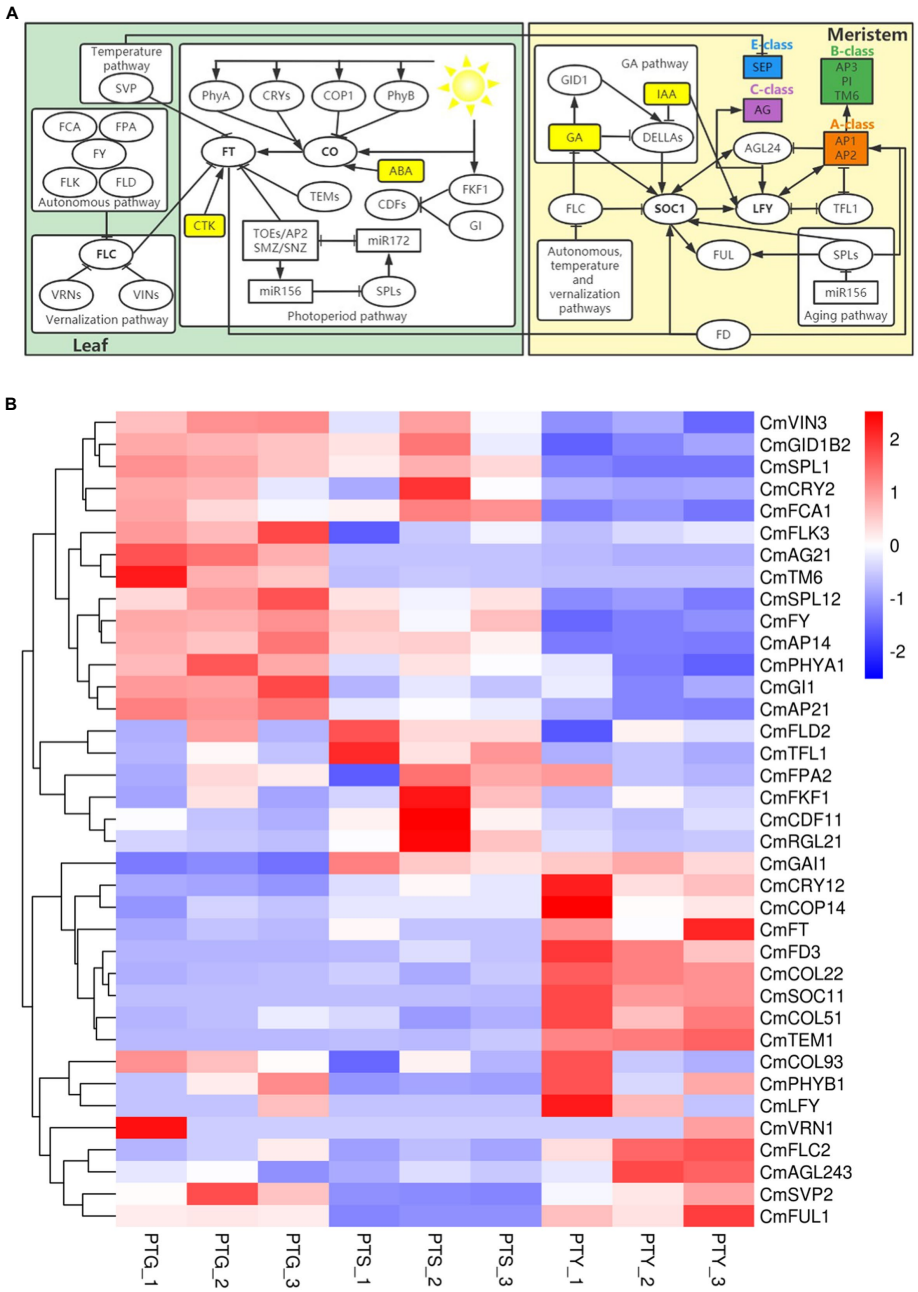
Important genes and signaling pathways involved in flowering control in chrysanthemum. **(A)** Schematic diagram of the flowering regulatory networks involved in six major floral induction pathways the *Chrysanthemum morifolium*. Arrows indicate activation. Bars indicate repression. **(B)** Heat map of the gene expression levels in the six major floral induction pathways. Rows and columns represent genes and samples, respectively. Sample names are provided below the heat map (PTS, ray florets; PTG, disc florets; PTY, leaves). The color scale indicates gene expression fold-changes (red, high expression; blue, low expression). All homologs of the regulators involved in the flowering regulatory networks are listed in Supplementary File 6.

Previous studies have identified six major floral induction pathways in *A. thaliana*, namely the ambient temperature, vernalization, autonomous, photoperiod pathway, GA, and age pathways (Sumitomo et al., 2009; Fornara et al., 2010; Gao et al., 2017; Wei et al., 2017; Oda et al., 2020). In this study, we identified many genes that were homologs of flowering time control genes involved in these floral induction signaling pathways. The expression levels of some of these genes are shown in Figure 6. Chrysanthemum floral induction is controlled mainly through the photoperiod and circadian signaling pathways by important flowering regulator genes, including *PHYTOCHROME* (*PHY*), *CRY*, *CONSTITUTIVE PHOTOMORPHOGENIC 1* (*COP1*), *CO*, *FT*, *TYPE B-LACTAMASE* (*TEM*), *CATION DIFFUSION FACILITATOR* (*CDF*), *LATEGIGANTEA* (*GI*), and *FLAVIN-BINDING KELCH REPEAT F-box1* (*FKF1*; Yang et al., 2017; Sun et al., 2018; Oda et al., 2020). We identified homologs of four *PHY*, three *CRY*, four *COP1*, 10 *CO*, one *FT*, one *TEM*, six *CDF*, four *GI*, and one *FKF1* in the chrysanthemum transcriptome data.

The ambient temperature pathway converges to regulate the expression of *FT* by *SVP*, and two homologs of *SVP* (*CmSVP1* and *CmSVP2*) were identified. Many autonomous pathway genes have been found in Arabidopsis, including *FLOWERING LOCUS CA* (*FCA*), *FLOWERING LOCUS PA* (*FPA*), *FLOWERING LOCUS D* (*FLD*), *FLOWERING LOCUS Y* (*FY*), and *FLOWERING LOCUS KH DOMAIN* (*FLK*; Eom et al., 2017). These genes promote flowering by inhibiting the expression of *FLOWERING LOCUS C* (*FLC*; Yan et al., 2020). We identified homologs of three *FCA*, four *FPA*, three *FLK*, three *FLD*, and one *FY* in chrysanthemum. FLC is a MADS-box TF that acts as a potent repressor of flowering in the vernalization pathway of different *Arabidopsis* varieties (Fornara et al., 2010). We detected homologs of two *FLC* (*CmFLC*1 and *CmFLC*2), one *VERNALIZATION INSENSITIVE 3* (*VIN3*) and one *VERNALIZATION 2* (*VRN2*) in this pathway. In the age pathway, SQUAMOSA PROMOTER BINDING LIKE (SPL) TFs promote flowering by initiating the expression of other TFs, such as LEAFY (LFY), FRUITFULL (FUL), and SOC1 (Fornara et al., 2010). We identified 19 homologs of *SPL* genes in this pathway.

The GA pathway converges to regulate *SOC1*, and we identified homologs of three *GAINSENSITIVE DWARF1* (*GID1*) and 15 *DELLA* in this pathway. We also found homologs of 91 auxin (IAA), 16 cytokinin (CTK), and 11 abscisic acid (ABA), and identified homologs of *LFY*, *FUL*, *TFL1* (*TERMINAL FLOWER 1*), *AGAMOUS like-24* (*AGL24*), and bZIP transcription factor FD genes. Details of all the identified homologs of the regulators in the flowering regulatory networks involved in the six major floral induction pathways of *C. morifolium* are listed in Supplementary File 6. On the basis of the annotations of the transcriptome sequences, homologs of the Class A, B, C, and E genes were identified, including homologs of four *AP1*, four *AP2*, three *SOC1*, two *AG2*, and one *TM6* (Supplementary File 6).

### Verification of transcriptome accuracy *via* a quantitative real-time polymerase chain reaction analysis

**Figure 7:**
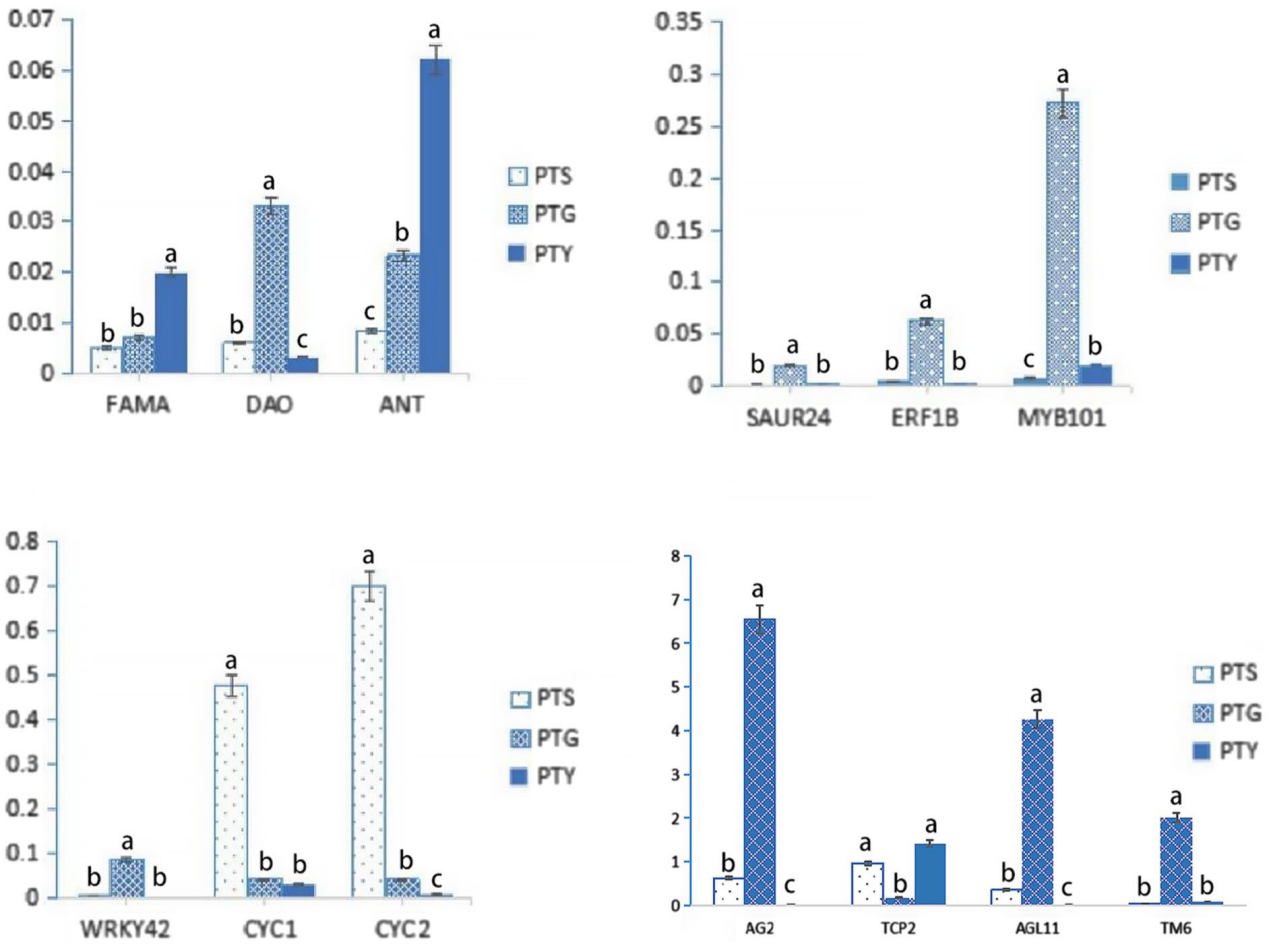
Relative expression levels of 13 floral development-related genes identified in the transcriptome data. Bars indicate standard error. The levels of each gene from the different samples (PTS, ray florets; PTG, disc florets; PTY, leaves) that are marked with the same letter were not significantly different.

The FPKM values indicated that *FAMA*, *DIAMINE OXIDASE* (*DAO*), *AINTEGUMENTA* (*ANT*), *SMALL AUXIN UP RNA24* (*SAUR24*), *ETHYLENE RESPONSE FACTOR 1B* (*ERF1B*), *MYB101*, *WRKY42*, *AG2*, and *AGAMOUS like-11* (*AGL11*), which are plant growth and development and floral development-related genes (Ohashi-Ito and Bergmann, 2006; Park et al., 2007; Jabbour et al., 2015; Ocarez and Mejía, 2016; Vuosku et al., 2019; Hara-Kitagawa et al., 2020; Niu et al., 2020; Zhang et al., 2020; Juárez-Corona and Stefan, 2021), as well as *CYC* and *TCP2*, which are flowering-related TF genes (Huang and Irish, 2015; Victoria and Minsung, 2017), are highly expressed in ray florets. *CYC* genes include *CYC1*, *CYC2*, and *CYC3* (Howarth and Donoghue, 2006). *CYC* and *TCP2* are also flower development-related genes (Juntheikki-Palovaara et al., 2014; Li et al., 2021). *TM6*, a member of MAD-box family, are highly expressed in disc florets, has proved determining the identity of stamen in plants (Tsaftaris et al., 2006; Yeoh et al., 2016; Martín-Pizarro et al., 2018). The expression levels of these genes were verified by qRT-PCR analysis. The results showed that *FAMA*, *ANT*, and *TCP2* were highly expressed in leaves, *DAO*, *SAUR24*, *ERF1B*, *MYB101*, *WRKY42*, *AG2*, *AGL11*, and *TM6* were highly expressed in disc florets, and *CYC1* and *CYC2* were highly expressed in ray florets. These results are consistent with the FPKM values obtained from the transcriptome data (Figure 7; Table 3), which confirms the reliability of the transcriptome sequencing results. Relative expression levels were determined using the PP2Ac-encoding gene as a internal control gene (Ren et al., 2013; Liu et al., 2015, 2016a).

### Analysis of the *CmTM6-mu* sequence

**Figure 8:**
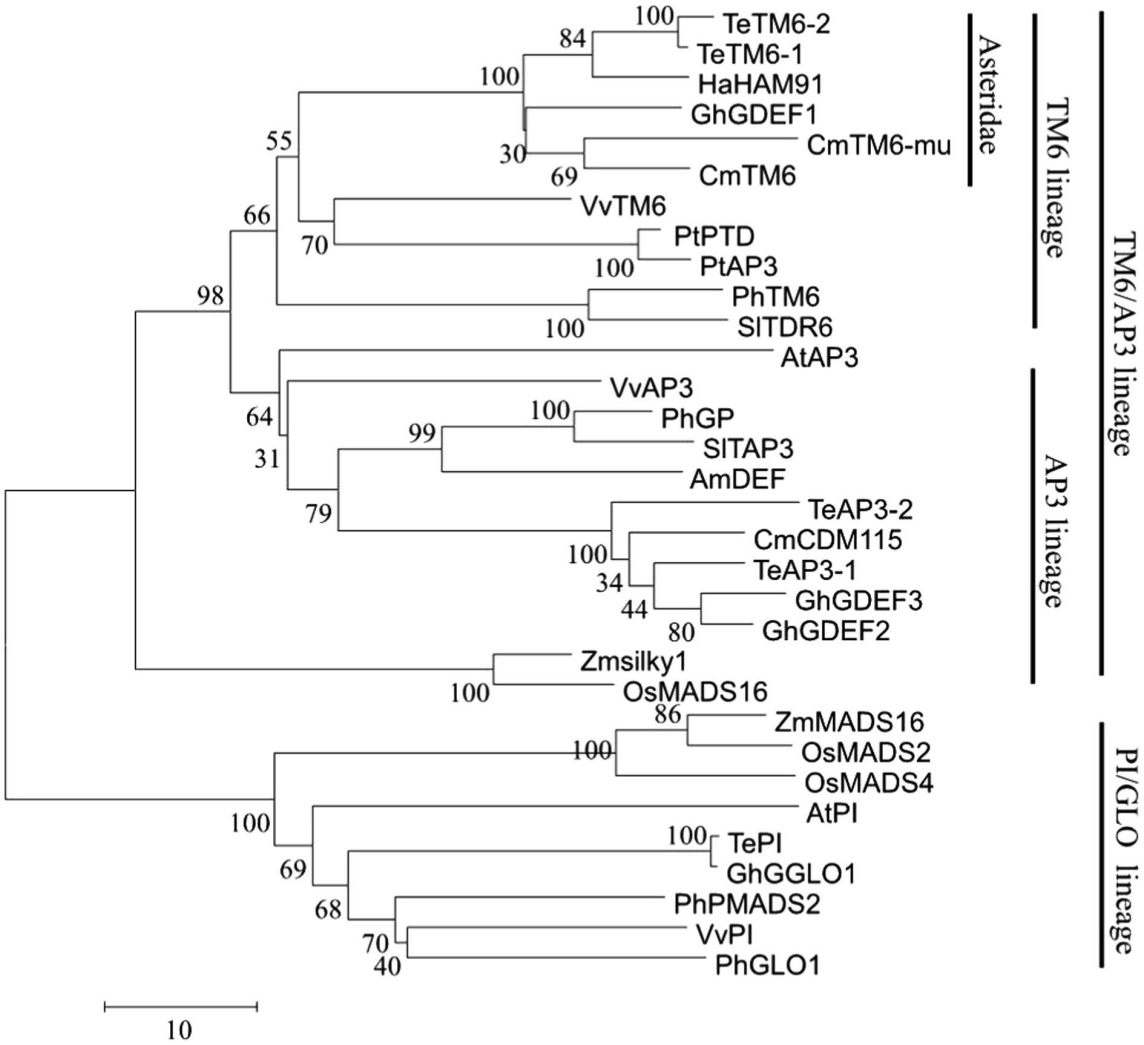
Phylogenetic tree with TM6/AP3 lineage and PI/GLO lineage proteins.

**Figure 9:**
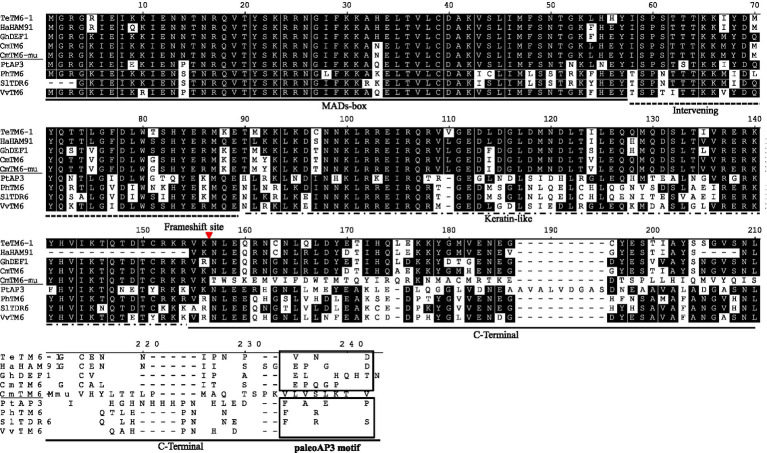
Alignment of the protein sequences encoded by *TM6* homologs in different organisms.

In the transcription database, a PTG-specific expression gene, which was annotated as *GDEF* and belongs to Class B floral homeotic MADS-box TF family, attracted our attention. This may be a key function gene related to disc florets development. This gene was isolated from *C. morifolium via* RT-PCR using primers designed on the basis of the transcription data. The amplified gene was 690 bp in length and was predicted to encode a protein comprising 229 amino acid residues. The coding product shared a high similarity (96.79%) with *Artemisia annua* GDEF1 protein (GenBank No. PWA78933) as determined by a BLAST search of the NCBI database. In the phylogenetic tree constructed using AP3 and PI proteins from different plants, the protein encoded by this gene clustered with TM6 lineage proteins (Figure 8; Supplementary File 5). A sequence alignment revealed that the encoded protein was highly similar to AP3-type proteins from other plants (Tsaftaris et al., 2006; Ackerman et al., 2008; Zhang et al., 2011), but the paleoAP3 motif typical of TM6 proteins was not present in the C-terminal region. A single-base deletion (one A base missing) was discovered at the 3′ end (Supplementary File 4), which resulted in a frameshift in the ORF that caused the final 70 amino acid residues at the C-terminal to be replaced with a different 73-amino acid sequence. Hence, this gene was designated as *CmTM6-mu*. The full *CmTM6* gene (i.e., without the deletion) encodes a protein with the expected C-terminal paleoAP3 motif (Figure 9).

**Table 5:** Specific primers for TM6 gene cloning.

Primer names	Primer sequences	Function	Tm (°C)	Length (bp)
TM6-1	ATACATTTGGCTGGTTTC	Nested PCR primer for *TM6* gene cloning	43.7	836
TM6-2	TTACCACCTAGTTAGTCTGTT			
TM6-3	AGAGATGGGTAGGGGTAAGAT		48	741
TM6-4	TCATTAATCACCTACGCAGC				

### Ectopic expression of *CmTM6-mu* in *Arabidopsis thaliana* induces early flowering

**Figure 10:**
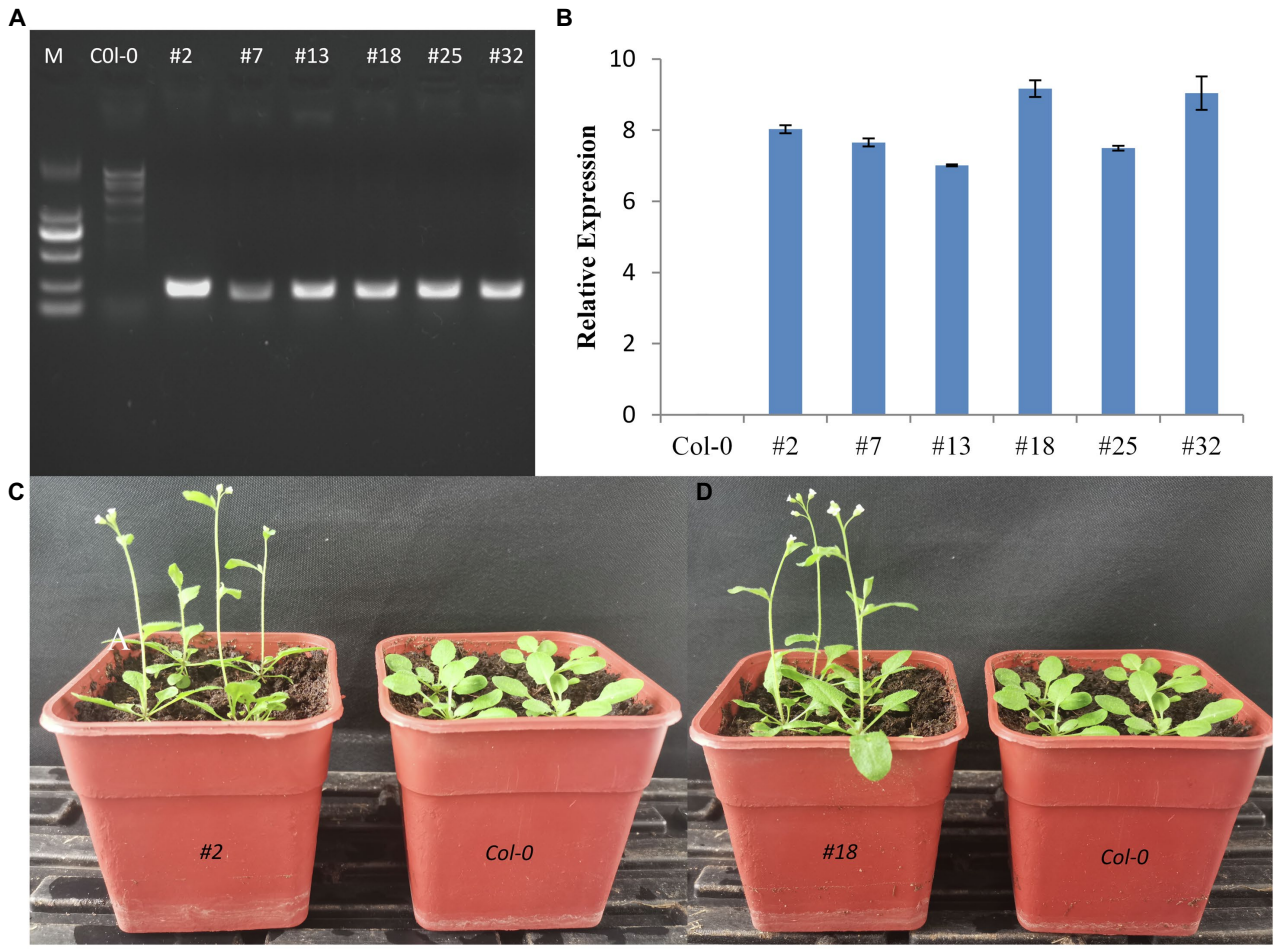
Effect of the ectopic expression of *CmTM6-mu* in *Arabidopsis thaliana*. **(A)** Detection of transgenic *A. thaliana* by PCR. **(B)** Relative gene expression levels in the transgenic *Arabidopsis* lines. **(C,D)** Phenotypes of wild-type Col-0 plants and *Arabidopsis* lines #2 and #18 overexpressing *CmTM6-mu*. respectively.

The *35S*:*CmTM6-mu* construct was inserted into *A. thaliana via* an *Agrobacterium tumefaciens*-mediated transformation method (Clough and Bent, 2010). We obtained 34 independent transgenic lines of 35S:*CmTM6-mu*, and nine transgenic lines were randomly selected for further analysis. Three weeks after sowing, the transgene expression levels in leaves of T3 generation *Arabidopsis* plants were analyzed by qRT-PCR. In all the tested transgenic lines, the *TM6-mu* genes were highly expressed; the highest *TM6-mu* expression level was detected in line #18 (Figures 10A,B). No distinguishable differences were found between transgenic *Arabidopsis* and wild-type *Arabidopsis* (Col-0) plants by morphological observation. However, seven of the nine transgenic lines produced earlier flowers than Col-0 *Arabidopsis* did (Figures 10C,D). The 35S:*CmTM6-mu* transgenic *Arabidopsis* lines began to bolt at ~23 days after sowing, whereas the wild-type Col-0 plants started to bolt at ~30 days after sowing, indicating that the transgenic lines flowered 5–7 days earlier than the wild-type plants (Figures 10C,D). The numbers of rosette leaves on the transgenic lines were lower (7 ± 1) than the numbers on the wild-type plants (11 ± 1). These results imply that *TM6-mu* had no effect on petal and stamen development, but affecting the flowering time in the transgenic *Arabidopsis* plants.

## Discussion

### Many important TFs (e.g., TCP, MYB, and MADS) that were differentially expressed between ray and disc florets may be important candidate genes for the floral development regulation mechanism in chrysanthemum

The whole transcriptome analysis of the DEGs between the ray and disc florets of chrysanthemum reflected the complexity of the regulatory machinery underlying the development of the capitula. The CYC and MADS TF families, as well as TFs from other families, have been shown to have key regulatory functions that influence floral development (Kaufmann et al., 2009; Hileman, 2014; Li et al., 2021). In this study, many floral development TF genes from the TCP, MYB, MADS, and other TF families (e.g., *AG2*, *TCP2*, *CYC1*, *CYC2*, *ANT*) were differentially expressed between the two floret types.

Earlier research on *CYC* genes in the model plant snapdragon proved the importance of these genes as regulators of floral symmetry (Clark and Coen, 2002; Galego and Almeida, 2002). The *CYC* genes also have crucial functions in sunflower, as well as in *Gerbera* and *Senecio* species (Burke, 2008; Kim et al., 2008; Chapman et al., 2012; Juntheikki-Palovaara et al., 2014). Moreover, studies on sunflower and *Gerbera* species, as well as chamomile, proved that *CYC* genes help regulate the formation of the two floret types in Compositae species (Broholm et al., 2008; Fambrini et al., 2011; Mizzotti et al., 2015; Wen et al., 2019). In our study, the qRT-PCR data were consistent with the FPKM values from the transcriptome data analysis, which confirmed that *CYC1* and *CYC2* were highly expressed in ray florets and lowly expressed in disc florets (Figure 7; Table 3). Thus, *CYC* genes are excellent candidates for studying chrysanthemum floral development.

In addition to *CYC* genes, floral organ-determining genes (e.g., *DIV*, *MYB*, and *AG2* genes) were differentially expressed in the two floret types. *MYB101*, *AG2*, and *AGL11* were highly expressed in disc florets, whereas *TCP2* was highly expressed in ray florets (Figure 7; Table 3). Most TCP family genes (e.g., *TCP2*) and *CYC* genes (*CYC1*, *CYC2*) were significantly more highly expressed in the ray florets than they were in the disc florets, whereas the AG family genes (*AG2*, *AGL11*) were significantly more highly expressed in disc florets than they were in ray florets. Besides, some AP, MYB, DIV, and MADS family genes were more highly expressed in ray florets than in disc florets, whereas others were more highly expressed in disc florets. These genes likely play important roles in chrysanthemum floret development, and therefore changes in their expression levels may cause meristems to differentiate in different directions. These genes need to be investigated more thoroughly to confirm their effects on chrysanthemum floret development.

### Many genes involved in flowering regulatory signaling pathways were identified in chrysanthemum, among them *CmTM6* was specifically expressed in disc florets

Flowering is a complex process that is controlled by environmental conditions and developmental regulation. Several flowering regulatory signaling pathways have been identified in *A. thaliana*, including the ambient temperature, vernalization, autonomous, photoperiod, GA, and age pathways (Sumitomo et al., 2009; Fornara et al., 2010; Gao et al., 2017; Wei et al., 2017; Oda et al., 2020). These different signaling pathways converge on important cross-regulatory genes, such as *FLC*, *SOC1*, *FT*, *CO*, and *LFY*. We outlined the gene regulatory networks involved in these pathways (Figure 6A) and identified homologs of the key regulators in chrysanthemum (Supplementary File 6). *FLC* encodes a MADS-box TF that inhibits flowering, and its expression suppressed flowering in the ambient temperature, autonomous, and vernalization pathways (Fornara et al., 2010; Liu et al., 2016a). We identified two homologs of *FLC* (*CmFLC1* and *CmFLC2*) in chrysanthemum. *FLC* inhibits flowering by repressing the expression of *SOC1* and *FT*, which are the early targets of *CO*, a key regulatory gene in the photoperiod pathway. *LFY* is a downstream regulatory gene of *SOC1* and a key regulator in the specification of floral meristem identity. *LFY* expression leads to a cascade of transcriptional activities that sustain indeterminate growth of the inflorescence meristem (Irish, 2010). On the basis of the protein annotation of the unigenes, we identified homologs of *SOC1* (*CmSOC11*, *CmSOC12*, *CmSOC13*), *FT* (*CmFT*), *CO* (*CmCOL91*, *CmCOL21*, *CmCOL22*, *CmCOL1*, *CmCOL92*, *CmCOL51*, *CmCOL93*, *CmCOL23*, *CmCOL94*, *CmCOL52*), and *LFY* (*CmLFY*).

Plant hormones (e.g., IAA, CTK, GA, ABA) are important endogenous signal participants that play important roles in the flowering process. The mechanisms of hormones in photoperiod and epigenetic regulation have been elucidated (Oda et al., 2017). Synergistic and antagonistic effects of various hormones have been found, and various hormones have been shown to be involved in flower formation regulation pathways mediated by DELLA proteins in the GA pathway (Sumitomo et al., 2009; Gao et al., 2017; Wei et al., 2017). In this study, we identified three homologs of *GID1* (*CmGID1B1*, *CmGID1B2*, *CmGID1A*) and 15 homologs of *DELLA* (e.g., *CmGAI1, CmGAI*, *CmRGL3*) in the GA pathway. IAA was the first plant hormone to be identified, and its involvement in the elongation and differentiation of plant cells, seed development, lateral root formation, root and leaf growth, development, and other physiological processes has been widely reported (Dinesh et al., 2016). IAA participates in GA biosynthesis and signal transduction by inhibiting the expression of *DELLA* (Fu and Harberd, 2003). We identified 91 homologs of IAA (e.g., *CmARF3*, *CmSAUR50*, *CmIAA1*) in flowering regulatory pathways. CTK regulates the division and differentiation of floral meristem cells, down-regulates miR172 expression levels, and promotes *AP2* expression (Aukerman and Sakai, 2003; Meijón et al., 2011); however, the mechanism of CTK in floral regulation has not been fully explained. We identified 16 homologs of CTK (e.g., *CmLOG5*, *CmCKX1*, *CmCKX3*) were found in chrysanthemum. Riboni et al. found that ABA activates the expression of *FT* and *TSF* to promote *Arabidopsis* flower bud differentiation (Riboni et al., 2013, 2016). In the ABA signaling network, ABA activates the ABA-response element binding protein (AREB) by promoting *CO* transcription, ABA insensitive 3 (ABI3) was ubiquitinated by ABA, ABI3-binding CO protein was released to promote flower formation (Zhang et al., 2005; Riboni et al., 2013, 2016). In this study, we identified 11 homologs of ABA (e.g., *CmCAR4*, *CmCAR4*, *CmAIB*) in chrysanthemum.

The Class A, B, C, and E genes specify flower organ identity. Class A genes include *AP1* and *AP2* (Mahajan and Yadav, 2014; Sasaki et al., 2014; Liu et al., 2016a; Prunet et al., 2017). *LFY* was shown to directly activate *AP1* transcription of Wagner et al. (1999). We identified four homologs of *AP1* (*CmAP11*, *CmAP12*, *CmAP13*, *CmAP14*) and four homologs of *AP2* (*CmAP21*, *CmAP22*, *CmAP23*, *CmAP24*). In most higher plants, Class B genes are divided into three lineages: *PI*, *euAP3*, and *TM6*. (Vandenbussche et al., 2003; Kramer et al., 2006; Ackerman et al., 2008). In this study, several Class B MADS-box genes were identified, and one gene (Cse_sc000306.1) was specifically expressed in disc florets (Table 3; Figure 7). This Class B MADS-box gene was identified as a homolog of the *TM6* lineage gene based on the encoded protein sequence and the evolutionary tree that contained all the functional Class B MADS-box genes (Figures 7, 8). In plants, the *PI/GLO* and *AP3*/*DEF*/*TM6* genes (i.e., Class B genes) control petal and stamen development (Kramer et al., 1998; Vandenbussche et al., 2003; Zhang et al., 2011). *PI/GLO* and *AP3/DEF*/*TM6* genes form separate lineages that arose from a duplication event that occurred ~260 million years ago (231–290 million years ago; Kim et al., 2004). Following the divergence of the *AP3/DEF/TM6* and *PI/GLO* lineages, many other duplication events occurred, including one that may be related to the separation of the *AP3* lineage from the ancestral *TM6* lineage in higher eudicots (Kramer et al., 2006; Broholm et al., 2008). In the C-termini of proteins encoded by the *AP3* lineage genes, the paleoAP3 motif has been replaced by the euAP3 motif (Martino et al., 2006; Rijpkema et al., 2006; Cao et al., 2019).

There has been substantial research on the derivation of the *euAP3* gene from the *paleoAP3* gene, as well as on the mechanism underlying gene functions and expression patterns (Kramer et al., 1998, 2006; Vandenbussche et al., 2003; Zhang et al., 2011). Initially, a new copy of the *paleoAP3* gene was produced *via* a duplication event, after which a frameshift due to a mutation resulted in the paleoAP3 motif at the C-terminus of the encoded protein being replaced by a novel euAP3 motif (Kramer et al., 1998; Zhang et al., 2011). This motif change coincided with the functional differentiation between the proteins (Kramer et al., 1998, 2006). During or after the duplication event that resulted in the *euAP3* and *TM6* lineages, an important change in the regulatory elements modulated the expression patterns of these genes (Kramer et al., 1998, 2006; Vandenbussche et al., 2003; Zhang et al., 2011). Finally, new gene lineages that varied in terms of functions and expression patterns were created (Kramer et al., 1998, 2006; Vandenbussche et al., 2003; Zhang et al., 2011). Along with gene duplications, gene losses also occurred in some plants, which included the loss of the *TM6* lineage genes in *A. majus* and *A. thaliana* (Lamb and Irish, 2003; Rijpkema et al., 2006). However, *AP3* and *TM6* coexist in most higher eudicots (Vandenbussche et al., 2003; Kramer et al., 2006; Tsaftaris et al., 2006; Ackerman et al., 2008). *AP3* and *TM6* genes have been isolated from plants in the Asteraceae family, such as *Gerbera hybrida* and sunflower (*Helianthus annuus*; Shulga et al., 2008; Broholm et al., 2010). Two *euAP3* type homologous genes, *CMD19* and *CDM115*, were isolated from chrysanthemum (Shchennikova et al., 2004, 2018), and in this study, we isolated a *TM6* type gene from chrysanthemum for the first time.

The *euAP3* genes, such as *AP3* in *A. thaliana* and *DEF* in *A. majus*, determine petal and stamen development, whereas *paleoAP3* lineage genes (*TM6*) affect only stamen development (Martino et al., 2006; Rijpkema et al., 2006; Cao et al., 2019). In petunia, four Class B genes have been found, including *euAP3* lineage *PhDEF* and *paleoAP3* lineage *PhTM6* (Rijpkema et al., 2006); *PhDEF* expression occurred in whorls 2 and 3, whereas *PhTM6* expression occurred mainly in whorls 3 and 4 (Rijpkema et al., 2006). Heterologous *paleoAP3* genes were able to rescue stamen development in the *Arabidopsis ap3* mutant that had both petal and stamen abnormalities (Fang et al., 2014; Jing et al., 2014), which is consistent with the observation that *paleoAP3* only affects stamen development. However, overexpression of heterologous *paleoAP3* genes in WT model plants seemed to have no phenotypic effects (Zhang et al., 2011; Ai et al., 2017), possibly because there are redundant functional genes in the receiving plants (Fang et al., 2014; Ai et al., 2017). In this study, the *TM6* gene in chrysanthemum was specially expressed in disc florets, and its expression was not detected in ray florets and leaves (Table 3; Figure 7). Besides the difference in symmetry, the most significant difference between ray and disc florets is that ray florets have no stamen, whereas disc florets have normal functional stamen. The gene expression patterns that we found in chrysanthemum flowers combined with similar findings reported previously in other plants, support the hypothesis that *TM6* acts as the trigger for stamen morphogenesis in chrysanthemum. The absence of stamen in ray florets, which are located in the outer side of the capitulum of chrysanthemum, may be linked to the non-expression of *TM6* in ray florets. However, to understand why the expression of the *TM6* gene is different between ray and disc florets requires further studies into the complex regulatory network that is involved.

### Frameshift in the chrysanthemum *TM6* gene regulated flowering time changes in *Arabidopsis*, indicating that *CmTM6-mu*, which has a new C-terminal motif, may have novel functions in the regulation of flowering time

We isolated a non-typical *TM6* lineage gene from *C. morifolium* flowers and named it *CmTM6-mu*. Phylogenetic analysis revealed that the encoded CmTM6-mu protein shared high similarity with TM6 proteins from other plants (Figure 8). The CmTM6-mu protein contained the same conserved MADS (M), intervening (I), and keratin-like (K) domains as other TM6 proteins, but had a completely different C-terminal that lacked the characteristic paleoAP3 motif found in other TM6 proteins (Figure 9). Further analyses detected a single-base deletion that cause a frameshift that changed the C-terminal sequence of the encoded CmTM6-mu protein (Supplementary File 4). Because AP3 and TM6 proteins have the same origin they share high similarity; both of them have conserved M, I, and K domains and variable C-terminals. All the conserved domains are necessary for their function. The M domain is highly conserved among the AP3, TM6, PI, and GLO proteins, and is important for binding to DNA by recognizing CArG sites, as well as acting as a mediator in protein dimerization (Pellegrini et al., 1995; Shore and Sharrocks, 1995; Hassler and Richmond, 2001). The function of the K domain is still unclear. In *Arabidopsis*, the K domain may mediate the strength and specificity of AP3/PI heterodimerization (Yang et al., 2003). The I domain is located between the M and K domains, and varies in length. The I domain is also necessary for DNA-binding and interactions with other proteins (i.e., heterodimer formation; Riechmann and Meyerowitz, 1997; Yang et al., 2003). The function of the C-terminal of AP3 is also unclear. Analyses of the phenotypes that resulted from ectopic gene expression and rescue experiments involving truncated proteins (i.e., removal of characteristic C-terminal motifs) showed that the C-terminal AtAP3 motif conferred AP3 functionality on the heterologous PI protein (Lamb and Irish, 2003). The C-terminal region is the only significantly different region between AP3 and TM6, which suggests the C-terminus may be responsible for the functional diversity between these proteins (Riechmann and Meyerowitz, 1997; Yang et al., 2003; Tzeng et al., 2004). The conserved M, I, and K domains of CmTM6-mu indicate that it may still have DNA-binding ability and the ability to interact with other proteins. The functions of these domains have not been studied in chrysanthemum, but similarities between the heterologous proteins AP3 (CMD115) and PI (CMD86) have been reported (Shchennikova et al., 2018).

Studies have shown that AP3 functions as a petal and stamen determiner, whereas TM6 only affects stamen development (Fang et al., 2014; Jing et al., 2014). We found that *CmTM6* was highly expressed in disc florets (with normal stamen), whereas its expression was almost undetectable in ray florets (stamens abortion) and in leaves, indicating that CmTM6 may be involved in stamen development. However, we do not know if the function of CmTM6-mu, which contains the conserved domains but has a mutation in the C-terminal, has changed. When the 35S:*CmTM6-mu* construct was transformed into *Arabidopsis*, an interesting and unexpected floral organ phenotype was observed in the transgenic plants, and earlier flowering occurred in almost all the transgenic lines. Similar results have been reported in other studies. An et al. found that ectopic expression in tobacco of a poplar *PtAP3* gene that had a non-conserved paleoAP3 motif caused early flowering (An et al., 2011). Ectopic expression in *Arabidopsis* of lily *LMADS1*, a paleoAP3 type gene that shares high homology with AP3 lineage proteins in monocots, was also shown to cause early flowering (Tzeng and Yang, 2001). In chrysanthemum, ectopic expression of a sunflower *TM6* lineage gene, *HAM91*, led to later flowering than in the WT (Shchennikova et al., 2018). Although, the mechanism of florescence change caused by TM6 is not clear, this finding still provides a new insight into the mechanism of flowering regulation in plants that can be clarified in future studies. Given the way that euAP3 originated from paleoAP3, it is possible that CmTM6-mu with the new C-terminal motif may have novel functions related to the regulation of flowering time. Many more studies are needed to confirm these findings.

## Conclusion

To screen for key genes that regulate the differentiation of the two types of chrysanthemum florets, we used leaves as a control for a transcriptome sequencing analysis of ray and disc florets. The generated data were analyzed to identify DEGs related to floret differentiation and development. The expression levels and patterns of the DEGs were verified by qRT-PCR analysis. The data presented herein suggest that *CYC* and *MADS* genes are involved in regulating the differentiation of the two types of chrysanthemum florets. Important regulatory genes and signaling pathways involved in flowering control were identified. Among them, a *TM6* gene (*CmTM6-mu*) that was specifically expressed in disc florets was found to be highly similar to other typical *TM6* lineage genes. Interestingly, a single-base deletion at the 3′ end of the *TM6* ORF resulted in a frame shift that caused the paleoAP3 motif to be missing at the C terminus of the encoded protein. Overexpression of *CmTM6-mu* in *Arabidopsis* led to earlier flowering of the transgenic lines, which indicated that *CmTM6-mu* with the new C-terminal motif may have novel functions in the regulation of flowering time. The results of this study provide valuable genomic information and candidate genes that will be useful for studies of flowering molecular mechanisms and for the breeding of novel flower types in chrysanthemums.

## Materials and methods

### Plant materials and RNA extraction

The ground-cover chrysanthemum variety *C. morifolium* Ramat “Pink Carpet” grown at the Shunyi Base of the Biotechnology Research Center of the Beijing Academy of Agriculture and Forestry Sciences was used as the study material. Ray and disc florets of the capitula, as well as fully extended leaves of the “Pink Carpet” cultivar were collected during the full-bloom stage (Figures 1A–C), with three biological replicates for each sample type. The collected samples were immediately frozen in liquid nitrogen and transported to the laboratory for storage at −80°C. Total RNA was extracted from the frozen materials using the MiniBEST Plant RNA Extraction Kit (TaKaRa, Dalian, China). The NanoDrop ND2000 spectrophotometer (Thermo Fisher Scientific, Barrington, IL, United States) was used to quantify the extracted RNA and assess RNA quality.

### Library construction and transcriptome sequencing

The extracted RNA was treated with DNase to eliminate any residual genomic DNA, after which magnetic beads with oligo-(dT) were used to enrich the eukaryotic mRNA. The mRNA was broken into short fragments. The disrupted mRNA and random hexamer primers were used to synthesize first-strand cDNA. After synthesizing the second cDNA strand, the double-stranded cDNA was purified using a commercial kit (Promega, Madison, WI, United States). The purified cDNA underwent an end-repair step before a poly-A tail and a sequencing adapter were added. Following a size selection step, the sequences were amplified by PCR with 2 x Phanta Max Master Mix (Vazyme, Nanjing, China). The libraries were qualitatively analyzed using the Agilent 2,100 Bioanalyzer (Agilent, Santa Clara, CA, United States) before they were sequenced using the Illumina HiSeq™ 2,500 system (Illumina, San Diego, CA, United States), which generated 125 or 150 bp paired-end reads.

### Data analysis

The image data obtained from the Illumina sequencer were used to generate raw data (raw reads) after base calling. The FastQC program^1^ was used to analyze the base quality of the raw data, and the results were visualized using R statistical software.^2^ Low-quality sequences were eliminated and Trimmomatic was used to remove the linker sequence (Bolger et al., 2014). The reads in which the initial sequence had a base quality lower than 3 and the terminal sequence had a base quality lower than 20 were removed to obtain high-quality reads (clean reads). The retained sequencing data of all the samples were combined, and the pre-processing quality and base distribution statistics were recorded. The HISAT2 program (Kim et al., 2015) was used to align the clean reads to the *C. seticuspe* reference genome to obtain information regarding the positions of reads in the reference genome or genes, as well as sequence information unique to the analyzed chrysanthemum materials. Genome comparison results were used to quantify genes. Additionally, the FPKM method (Roberts et al., 2011) was used to analyze the expression of protein-coding genes. The number of reads (Mortazavi et al., 2008), the accuracy of the FPKM calculations, and the correlations between the sequencing results for the different samples, especially the biological replicates, were analyzed to ensure the sequencing data were reliable. The correlations between the protein-coding gene expression levels of the samples were also determined. Correlation coefficients close to 1 were indicative of highly similar expression patterns between samples. Genes differentially expressed between samples were identified using the DESeq software (Anders and Huber, 2010). The baseMean value was used to estimate expression levels. The fold-change in the expression levels was calculated, and the negative binomial distribution test was used to evaluate the significance of the change. When using RNA-seq data to determine whether a gene is differentially expressed in two samples, the following two factors are considered: the expression-level fold-change and the value of p or false discovery rate (FDR; i.e., adjusted value of *p*). To calculate the FDR, the value of *p* for the expression in each sample was determined, after which the FDR error control method was used for the multiple hypothesis test correction of the value of ps. The identified DEGs were functionally characterized on the basis of a GO enrichment analysis. Additionally, the KEGG database was used to determine the enriched metabolic pathways associated with the DEGs.

### Analysis of differentially expressed genes

The DEGs were screened according to the FPKM values for the unigenes in each sample. The DEGs were identified on the basis of an FPKM ratio between samples that was >2 or <0.5. To control false positives, Benjamini and Hochberg FDR multiple check values were used. The corrected value of *p* was <0.05. The ray florets and disc florets were compared to screen for DEGs between the two floret types.

### Gene expression analysis *via* qRT-PCR

Total RNA was extracted from the ray florets, disc florets, and leaves as described above. Total RNA was treated with DNase (Promega, Madison, WI, United States) and then reverse-transcribed to cDNA using a commercial reverse transcription system (Tiangen, China). The qRT-PCR analysis was performed using the PikoReal real-time PCR system (Thermo Fisher Scientific, Germany). Each reaction was completed in a total volume of 20 μl, which contained 2 μl first-strand cDNA as the template. The PCR program was as follows: 95°C for 30 s, 40 cycles of 95°C for 5 s and 60°C for 30 s. Details regarding the gene-specific qRT-PCR primers are provided in Table 4. The qRT-PCR experiments were conducted using three biological replicates. Each replicate was analyzed in triplicate. The relative expression levels were calculated according to the 2^−ΔΔCt^ method. The *C. morifolium* gene encoding protein phosphatase 2A (*PP2Ac*) was used as the internal control reference gene to determine relative expression levels.

**Table 4:** Primers for the quantitative real-time PCR analysis.

Gene	Forward primer sequences	Reverse primer sequences
*FAMA*	CTTGAAGTTTCCAGTGTTGA	CTTATTCTTGCCTTCGGTA
*DAO*	CAATGTAAAAGCAGGATAAAC	GCAATGAGAATAGAAAGGGT
*ANT*	GATGCTGCCGAGGCTTAT	TGTGGGCTCCTGGACTTT
*SAUR24*	TAAATAGGGGTTGTTCCAA	GCATCATTCGTCTTCCAT
*ERF1B*	GCCCATCAAAGTTAGCAC	AGATAAGGGATTCAACCAGA
*MYB101*	CATCATCAAATCACGCAAAC	AACATAGACGACGGGGAG
*WRKY42*	GCAAAAGATGGCGAAAGG	GCTGGTGGTAATGGGTGG
*CYC1*	ACTTTGGTGCTGCCTTCT	CCCTTGATTGGCTCTTTAC
*CYC2*	GCAAGTTCACTCCAAAAGC	TCAATCAAGGGCAGAGGC
*AG2*	CGCCTTTATGAGTATGCC	AGGTTTGCGATTTGTGAA
*TCP2*	ATCTTTACCACCCGAGCCT	TATCCAAGCGAAAACCCA
*AGL11*	CAAAGATCAGGTCCAAGAAGA	TGCATTATACGCATGTCCACT
*TM6*	AAATGGTAATCTTCGACTGGAC	TGGGTAATGTTGTTAGGTAGTGC
*PP2Ac*	ATCAGAACAGGAGGTCAGGG	TAATTTGTATCGGGGCACTT

### Molecular cloning of *CmTM6-mu*

Total RNA was extracted from chrysanthemum flowers using the MiniBEST Plant RNA Extraction Kit (TaKaRa). First-strand cDNA was synthesized using a reverse transcription system (Promega). Primers specific for the *TM6* ORF (Table 5) were designed on the basis of a sequence annotated as *TM6* in the transcriptome data for chrysanthemum flowers. The PrimeSTAR HS DNA polymerase kit (TaKaRa) was used for amplifying the *TM6* ORF. The PCR product was examined by 1% agarose gel electrophoresis. The amplified fragment purified from the agarose gel was inserted into the pGEM-T Easy vector (Promega) and sequenced by the Shanghai Sangon Company.

### Sequence alignment and phylogenetic analysis

Sequences were aligned using the DNAStar 5.0 software^3^ in accordance with the ClustalW method. A phylogenetic tree was constructed using the MEGA program (version 6.0; Tamura et al., 2013). Specifically, the neighbor-joining method was used, with 1,000 bootstrap replicates. The sequences of *TM6*, *PI*, and *AP3* homologs were obtained from the GenBank database^4^ (Supplementary File 5).

### Construction of the overexpression vector and transformation of *Arabidopsis thaliana*

The full-length *CmTM6-mu* cDNA sequence was amplified by PCR using gene-specific primers (Table 5) and then inserted downstream of the 35S promoter in the pCAMBIA1301 vector digested with HindIII and XbaI. The *35S:CmTM6-mu* construct in the recombinant plasmid was sequenced to confirm its accuracy before it was introduced into *A. tumefaciens* strain EHA105 cells *via* electroporation. The *A. tumefaciens*-mediated genetic transformation of *A. thaliana* Col-0 was performed in accordance with a published floral-dip method (Clough and Bent, 2010). The putative transgenic lines were selected on MS medium containing 40 mg/l hygromycin B. The hygromycin B-resistant seedlings (T0 generation) were transferred to soil and grown in a greenhouse at 22°C, with 70% relative humidity and a 16 h light/8 h dark cycle. The presence of the transgene in the transgenic *Arabidopsis* plants was confirmed by qRT-PCR using the *TM6* primers list in Table 4. Phenotypic analyses were performed using the independent T3 transgenic lines. Three biological replicates were used for both the qRT-PCR and phenotypic analyses.

## Data availability statement

The data presented in the study are deposited in the repositories of NCBI Sequence Read Archine with BioProject accession number: PRJNA821995 and SRA accession number: SRR18585549–SRR18585557. We confirm the data had been released on 2022-04-11.

## Author contributions

YJ, YC, SW, HC, XZ, SG, DC, and HL conducted the experiments. YC and SG analyzed the data and prepared the manuscript. DC and HL reviewed the manuscript. CH provided assistance. All authors contributed to the article and approved the submitted version.

## Funding

This research was supported by the Youth Fund of Beijing Academy of Agriculture and Forestry Sciences (QNJJ202010), National Natural Science Foundation of China (31901354), the Innovation Foundation of the Beijing Academy of Agriculture and Forestry Sciences (KJCX20200205), and Study on breeding technology and ecological adaptability of Lily for both ornament and food (KM202112448004). These funding bodies did not participate in the design of the study, collection, analysis, and interpretation of data or writing the manuscript.

## Conflict of interest

The authors declare that the research was conducted in the absence of any commercial or financial relationships that could be construed as a potential conflict of interest.

## Publisher’s note

All claims expressed in this article are solely those of the authors and do not necessarily represent those of their affiliated organizations, or those of the publisher, the editors and the reviewers. Any product that may be evaluated in this article, or claim that may be made by its manufacturer, is not guaranteed or endorsed by the publisher.

## Footnotes

^1^ https://www.bioinformatics.babraham.ac.uk/

^2^ https://www.statgraphics.com/statgraphics-r-interface

^3^ https://www.dnastar.com/

^4^ https://www.ncbi.nlm.nih.gov/

## Supplementary material

The Supplementary material for this article can be found online at: https://www.frontiersin.org/articles/10.3389/fpls. 2022.947331/full#supplementary-material

Supplementary File 1 Differentially expressed genes between disc florets and ray florets of *Chrysanthemum morifolium*.

Supplementary File 2 GO categories enriched with expressed genes between disc florets and ray florets of *Chrysanthemum morifolium*.

Supplementary File 3 KEGG pathways enriched with expressed genes between disc florets and ray florets of *Chrysanthemum morifolium*.

Supplementary File 4 Sequence and mutation information of CmTM6 and CmTM6-mu.

Supplementary File 5 Details of the proteins used to construct the phylogenetic tree.

Supplementary File 6 Homologous genes in the flowering regulatory networks involved in the six major floral induction pathways of *Chrysanthemum morifolium*.

## Abbreviations

DEG: Differentially expressed gene
FDR: False discovery rate
FPKM: Fragments per kilobase of transcript per million mapped reads
qRT-PCR: Quantitative real-time PCR
PCR: Polymerase chain reaction
TF: Transcription factor
