# Supplementary material for: Whole-transcriptome analysis of differentially expressed genes between ray and disc florets and identification of flowering regulatory genes in Chrysanthemum morifolium
Source: Front Plant Sci. 2022 Aug 4;13:947331. doi: 10.3389/fpls.2022.947331 (PMC9388166; doi:10.3389/fpls.2022.947331)
Supplement: Supplementary File 1 — Differentially expressed genes between disc florets and ray florets of Chrysanthemum morifolium. [file Table_1.DOCX]

1 ATGGGTAGGGGTAAGATAGAGATTAAGAAGATAGAAAATAATACAAACAGGCAGGTGACA

M G R G K I E I K K I E N N T N R Q V T

61 TATTCGAAGAGAAGAAATGGGATTTTCAAGAAAGCTAATGAGCTCACTGTTCTTTGTGAT

Y S K R R N G I F K K A N E L T V L C D

121 GCCAAAGTTTCACTTATTATGTTCTCTAACACTGGCAAATTACATGAGTACATTAGTCCT

A K V S L I M F S N T G K L H E Y I S P

181 TCGACAACGACTAAGAAGATGTATGACATGTATCAGACTACTGTAGGATTTGATCTGTGG

S T T T K K M Y D M Y Q T T V G F D L W

241 GGTTCCCACTATGAGAGGATGAAGGAAACGATGTACAAGCTCAAAGATACTAACAATAAA

G S H Y E R M K E T M Y K L K D T N N K

301 CTTAGGAGAGAGATCAGGCAAAGAGTTCTTGGTGAAGATATAGATGGTTTGGACATGAAT

L R R E I R Q R V L G E D I D G L D M N

361 GATCTCACAGTTCTCGAACAACAAATGCAAGATTCACTAACCGTTGTGCGAGAACGCAAG

D L T V L E Q Q M Q D S L T V V R E R K

421 TATCACGTGATCAAAACTCAGACCGACACTTGCAGGAAAAGGGTGAAAAACTTGGAGCAA

Y H V I K T Q T D T C R K R V K N L E Q

T W S K

481 AGAAATGGTAATCTTCGACTGGACTATGACACAATACATCAGGCAGAGAAAAAATATGGC

R N G N L R L D Y D T I H Q A E K K Y G

E M V I F D W T M T Q Y I R Q R K N M A

541 ATGCATGAGAACGAAGGAGGATACGAGTCCACTATTGCATATTCAAATGGTGTATCAAAT

M H E N E G G Y E S T I A Y S N G V S N

C M R T K E D T S P L L H I Q M V Y Q I

601 CTCTATGGCTTTTGTGCACTACCTAACAACATTACCCATGGCTCAGGGTACGAGCCCCAA

L Y G F C A L P N N I T H G S G Y E P Q

S M A F V H Y L T T L P M A Q G T S P K

661 GGTCCTCGTCTCGCTTAAGACTTTAGTTTAA

G P R L A * *CmTM6*

V L V S L K T L V * *CmTM6-mu*

Supplementary File 4 | Sequence and mutation information of *CmTM6* and *CmTM6-mu*.

Note: The deletion base was boxed; the stop codons are underlined.
