# Supplementary material for: Whole-transcriptome analysis of differentially expressed genes between ray and disc florets and identification of flowering regulatory genes in Chrysanthemum morifolium
Source: Front Plant Sci. 2022 Aug 4;13:947331. doi: 10.3389/fpls.2022.947331 (PMC9388166; doi:10.3389/fpls.2022.947331)
Supplement: Supplementary File 2 — GO categories enriched with expressed genes between disc florets and ray florets of Chrysanthemum morifolium. [file Table_2.DOCX]

| Name | NCBI NO. | lineage | Source (Latin name) |
| --- | --- | --- | --- |
| *GhGGLO1* | CAA08804 | PI/GLO lineage | *Gerbera hybrida* |
| *GhGDEF1* | CAA08802 | TM6 lineages |  |
| *GhGDEF2* | CAA08803 | AP3 lineage |  |
| *GhGDEF3* | ACV53813 | AP3 lineage |  |
| *TePI* | AOD74998 | PI/GLO lineage | *Tagetica erecta* |
| *TeAP3-1* | AOD74999 | AP3 lineage |  |
| *TeAP3-2* | AOD75000 | AP3 lineage |  |
| *TeTM6-1* | AOD75001 | TM6 lineage |  |
| *TeTM6-2* | AOD75002 | TM6 lineage |  |
| *CmCDM115* | AAO22985 | AP3 lineage | *Chrysanthemum x morifolium* |
| *HaHAM91* | AAO18231 | TM6 lineages | *Helianthus annuus* |
| *AtPI* | P48007 | PI/GLO lineage | *Arabidopsis thaliana* |
| *AtAP3* | AAA32740 | AP3 lineage |  |
| *AmDEF* | P23706 | AP3 lineage | *Antirrhinum malus* |
| *PhGP* | CAA39567 | AP3 lineage | *Petunia x hybrida* |
| *PhGLO1* | AAS46018 | PI/GLO lineage |  |
| *PhPMADS2* | CAA49568 | PI/GLO lineage |  |
| *PhTM6* | AF230704 | TM6 lineages |  |
| *OsMADS16* | AF077760 | AP3 lineage |  |
| *OsMADs2* | L37526 | PI/GLO lineage |  |
| *OsMADS4* | L37527 | PI/GLO lineage |  |
| *ZmMADS16* | NP_001105136 | PI/GLO lineage | *Solanum lycopersicum* |
| *ZmSilky1* | AAF59838 | AP3 lineage |  |
| *SlTDR6* | CAA43171 | TM6 lineages |  |
| *SlTAP3* | ABG73412 | AP3 lineage |  |
| *VvPI* | ABK59993 | PI/GLO lineage | *Vitis vinifera* |
| *VvTM6* | ABI98021 | TM6 lineage |  |
| *VvAP3* | EF418603 | AP3 lineage |  |
| *PtPTD* | AAC13695 | TM6 lineages | *Populus trichocarpa* |
| *PtAP3* | AY210488 | TM6 lineages |  |

Supplementary File 5 | Information regarding the proteins used for constructing the phylogenetic tree.
